# *De novo* and inherited private variants in *MAP1B* in periventricular nodular heterotopia

**DOI:** 10.1371/journal.pgen.1007281

**Published:** 2018-05-08

**Authors:** Erin L. Heinzen, Adam C. O'Neill, Xiaolin Zhu, Andrew S. Allen, Melanie Bahlo, Jamel Chelly, Ming Hui Chen, William B. Dobyns, Saskia Freytag, Renzo Guerrini, Richard J. Leventer, Annapurna Poduri, Stephen P. Robertson, Christopher A. Walsh, Mengqi Zhang, for the Epi4K Consortium, Epilepsy Phenome/Genome Project

**Affiliations:** 1 Institute for Genomic Medicine, Columbia University Medical Center, New York, New York, United States of America; 2 Department of Women’s and Children's Health, Dunedin School of Medicine, University of Otago, Dunedin, New Zealand; 3 Center for Statistical Genetics and Genomics, Duke University Medical Center, Durham, North Carolina, United States of America; 4 Department of Biostatistics and Bioinformatics, Duke University, Durham, North Carolina, United States of America; 5 Population Health and Immunity Division, The Walter and Eliza Hall Institute of Medical Research, Parkville, Victoria, Australia; 6 Department of Medical Biology, School of Mathematics and Statistics, University of Melbourne, Parkville, Victoria, Australia; 7 Pôle de Biologie, Hôpitaux Universitaires de Strasbourg, Strasbourg, France; 8 IGBMC, INSERM U964, CNRS UMR 7104, Université de Strasbourg, Illkirch, France; 9 Department of Cardiology and Division of Genetics and Genomics, Boston Children’s Hospital, Boston, Massachusetts, United States of America; 10 Departments of Pediatrics and Neurology, University of Washington, Seattle, Washington, United States of America; 11 Center for Integrative Brain Research, Seattle Children's Research Institute, Seattle, Washington, United States of America; 12 Department of Medical Biology, University of Melbourne, Parkville, Victoria, Australia; 13 Neuroscience Department, Children's Hospital Anna Meyer-University of Florence, Florence, Italy; 14 Department of Neurology Royal Children’s Hospital, University of Melbourne, Parkville, Victoria, Australia; 15 Murdoch Children’s Research Institute, University of Melbourne, Parkville, Victoria, Australia; 16 Department of Pediatrics, University of Melbourne, Parkville, Victoria, Australia; 17 Department of Neurology, Division of Epilepsy and Clinical Neurophysiology, Boston Children's Hospital, Boston, Massachusetts, United States of America; 18 Division of Genetics and Genomics, Manton Center for Orphan Disease Research and Howard Hughes Medical Institute, Boston Children’s Hospital, Boston, Massachusetts, United States of America; 19 Departments of Pediatrics and Neurology, Harvard Medical School, Boston, Massachusetts, United States of America; 20 Program in Medical and Population Genetics, Broad Institute of MIT and Harvard, Cambridge, Massachusetts, United States of America; 21 Program in Computational Biology and Bioinformatics, Duke University, Durham, NC, United States of America; Stanford School of Medicine, UNITED STATES

## Abstract

Periventricular nodular heterotopia (PVNH) is a malformation of cortical development commonly associated with epilepsy. We exome sequenced 202 individuals with sporadic PVNH to identify novel genetic risk loci. We first performed a trio-based analysis and identified 219 *de novo* variants. Although no novel genes were implicated in this initial analysis, PVNH cases were found overall to have a significant excess of nonsynonymous *de novo* variants in intolerant genes (p = 3.27x10^-7^), suggesting a role for rare new alleles in genes yet to be associated with the condition. Using a gene-level collapsing analysis comparing cases and controls, we identified a genome-wide significant signal driven by four ultra-rare loss-of-function heterozygous variants in *MAP1B*, including one *de novo* variant. In at least one instance, the *MAP1B* variant was inherited from a parent with previously undiagnosed PVNH. The PVNH was frontally predominant and associated with perisylvian polymicrogyria. These results implicate *MAP1B* in PVNH. More broadly, our findings suggest that detrimental mutations likely arising in immediately preceding generations with incomplete penetrance may also be responsible for some apparently sporadic diseases.

## Author summary

Almost 20 years ago the first gene responsible for periventricular nodular heterotopia (PVNH), a disorder that leads to abnormal migration of neurons during fetal brain development, was discovered. Since that time additional genes have been identified, but collectively they only explain a minority of cases. In this work we sought to further elucidate the genetic basis of this disorder using exome sequencing of 202 individuals with PVNH. We found a clear role for *de novo* mutations in PVNH, although with this analysis alone we were unable to pinpoint which of the *de novo* mutations in novel genes caused the disease. One patient was found to have a *de novo* variant in *MAP1B*, a gene that encodes a protein that plays a role at several key steps of brain development. With further analysis of the exome sequence data we found an additional three cases with a very rare inherited variant in *MAP1B*. This pattern is not expected to occur by chance and therefore indicates that these variants are likely responsible for the PVNH in these patients. Further strengthing the association of *MAP1B* in PVNH, all of the patients with a *MAP1B* variant had a similar brain abnormality, and at least one of the parents who transmitted the variant to their child was also similarly affected. This work adds to a growing list of genes responsible for PVNH, illuminates new genes involved in brain development, and importantly informs us about the types of genetic variants involved in PVNH.

## Introduction

Malformations of cortical development are phenotypically heterogeneous and frequently associated with epilepsy, intellectual disability and congenital neurological deficits [1]. Periventricular nodular heterotopia (PVNH) is one such malformation where a population of neurons fails to migrate to the cerebral cortex and instead adopt heterotopic positions along their sites of origin–adjacent to the lateral ventricles [2]. Ten loci [*FLNA*, *ARFGEF2*, *FAT4*, *DCHS1*, *EML1*, *NEDD4L*, *INTS8*, *EML1*, *AKT3*, *MCPH1* and *C6orf70* (also known as *ERMARD*)] are currently implicated in the causation of PVNH [3–11]. Variants in these genes explain approximately 25% of sporadic instances of the brain malformation, with variants in *FLNA* being the most frequently found [3–10]. Despite only a small number of genes identified to date, germline genetic variation is thought to explain a significant fraction of patients, particularly in light of the often bilateral symmetric presentation and the lack of evidence for extrinsic etiologies [12–14].

To further characterize the genetic bases of PVNH, we exome sequenced 202 trios with sporadic PVNH and performed two analyses (Methods). First, we executed a trio-based approach to search for *de novo* risk variants in the patient population. Given the phenotypic heterogeneity of PVNH, ranging from mild, sometimes subclinical, to very severe [15, 16], and the presence of X-linked *FLNA*-positive cases in both sporadic and inherited PVNH, we also sought to evaluate the role of risk alleles agnostic to the mode of inheritance by performing a gene-level case-control collapsing analysis of 196 probands (excluding six individuals sequenced from lymphoblastoid cell line DNA) and controls. In the collapsing analysis, we searched for enrichment of rare, putatively deleterious variants (inherited or *de novo*), within the protein-coding sequence of individual genes [17, 18]. The results of our analyses specifically pinpointed *de novo* and inherited variants in *MAP1B* in PVNH, and more broadly implicate ultra-rare, likely recently acquired variation in the genetic architecture of PVNH. Finally, given the challenges associated with distinguishing disease-relevant variations from background variation in genetically heterogeneous conditions, we utilized human brain-specific transcriptomic data [19, 20] to undertake a systems genetic analysis aimed at further organizing candidates for future investigation.

## Results

Given the prominent role for *de novo* variation in severe, sporadic neurodevelopmental disorders [21–26], we first identified *de novo* variants within trios using GATK multi-sample calling as described previously [21, 27]. A total of 219 *de novo* variants were identified in the 202 trios (1.1 per trio, Methods, S1 Table).

Among these *de novo* variants, nine were located in *FLNA*, a previously identified PVNH gene (Table 1)[3]. Consistent with the known role of *FLNA* in PVNH, observing nine *de novo* variants in *FLNA* is extremely unlikely to occur by chance (p = 3.4x10^-23^, FitDNM method[28]). A *de novo* variant was also identified in *NEDD4L*; the genetic findings in this same patient were previously reported in Broix et al [7]. No *de novo* variants were detected in *C6orf70* (also known as *ERMARD*), a previously identified dominant PVNH gene [6]. Three additional genes had multiple *de novo* variants in unrelated individuals (Table 1), including three *de novo* variants in *CHD5* and two each in *UGGT1* and *PLXNC1*. *CHD5* did not have a statistically significant excess of *de novo* variants in a cohort of this size compared to that expected based on the mutability and size of the gene, as assessed using the FitDNM method [28] (p = 1.6 x 10^−5^). Because we do not have estimates of mutation rates for insertion-deletion variants, and both *UGGT1* and *PLXNC1* harbored one single nucleotide substitution and one insertion-deletion variant, we were not able to formally test for enrichment of *de novo* variants in these genes using the FitDNM method.

**Table 1:** Genes with multiple *de novo* variants.

Gene	Proband ID	variant id (chr-position-ref-var, hg19)	ExAC v0.3.1 MAF	RefSeq Transcript ID	Function	CDS position	Protein position	Amino acid substitution	Polyphen2
*CHD5*	pvhnd29397ly1	1-6185252-G-A	0.00004	NM_015557.2	synonymous	4302	1434	N	-
*CHD5*	pvhcw8001bvg1	1-6219435-C-G	0	NM_015557.2	missense	348	116	K/N	benign
*CHD5*	pvhit130Lbou1	1-6166767-C-A	0	NM_015557.2	missense	5651	1884	R/L	probably_damaging
*FLNA*	pvhnd39214nu1	X-153592477-G-C	0	NM_001110556.1	stop_gained	2193	731	Y/*	-
*FLNA*	pvhcw14103bvj1	X-153590679-G-A	0	NM_001110556.1	stop_gained	2587	863	R/*	-
*FLNA*	pvhnd32846lz1	X-153590679-G-A	0	NM_001110556.1	stop_gained	2587	863	R/*	-
*FLNA*	pvhnd39654ajz1	X-153589918-G-A	0	NM_001110556.1	stop_gained	2965	989	Q/*	-
*FLNA*	pvhnd26332me1	X-153586868-G-A	0	NM_001110556.1	stop_gained	4543	1515	R/*	-
*FLNA*	pvhnd29397ly1	X-153593004-G-A	0	NM_001110556.1	stop_gained	1912	638	Q/*	-
*FLNA*	pvhnd25061mw1	X-153588557-C-CCCCG	0	NM_001110556.1	frameshift indel	3605–3606	1202	-	-
*FLNA*	pvhnd37807nj1	X-153599348-ATCT-A	0	NM_001110556.1	Inframe indel	263–265	88–89	KM/M	-
*FLNA*	pvhcw8001bvg1	X-153581372-C-A	0	NM_001456.3	missense	6199	2067	A/S	probably_damaging (0.986)
*PLXNC1*	pvhnd21601lu1	12-94658948-TAC-T	0	NM_005761.2	frameshift indel	3545–3546	1182	-	-
*PLXNC1*	pvhit256Jbpb1	12-94648646-C-T	0.00001	NM_005761.2	missense_variant	2965	989	R/W	probably_damaging (0.999)
*UGGT1*	pvhnd27930mf1	2-128890752-CG-C	0	NM_020120.3	Frameshift indel	1416	472	-	-
*UGGT1*	pvhnd40500bij1	2-128855098-A-C	0	NM_020120.3	missense	154	52	T/P	benign (0.366)

We next evaluated if *de novo* variation across the cohort had a distinct profile compared to controls using two orthogonal approaches. First, we performed a hot-zone analysis using previously described methods comparing profiles of *de novo* variation predicted to alter the level or activity of a protein that is encoded by a gene that has less than expected functional variation in the population (intolerant gene) in cases and controls [22] (Methods). We found significant enrichment of hot-zone *de novo* variants in PVNH cases (30.4%) compared to controls (9.6%) (p = 0.001). Removing the disease-causing *FLNA* variants from this analysis, a significant enrichment remained in PVNH cases (25%) compared to controls [odds ratio = 3.31 (95% CI: 1.22–9.08), p = 0.01]. Second, to further validate the observations from the hot-zone analysis comparing cases to controls, we also developed a likelihood model analysis to evaluate if the distribution of single nucleotide *de novo* variants in affected individuals differs significantly from that expected in the general population using a modified version of previously described methods [22, 27] (S1 Text). The model includes parameters estimating the relative risk associated with types of *de novo* variants and the proportion of the exome that confers PVNH risk. Given the results of the hot-zone analysis we specifically focused on analyses of the distribution of nonsynonymous *de novo* variants in the 4,317 intolerant genes [genes with a Residual Variation Intolerance Score (RIVS score) in the lowest 25 ^th^ percentile]. We observed a highly significant shift in the distribution from expectation (p = 3.27x10^-7^). Further, point estimates from the *de novo* mutation architecture model suggest that <1% of intolerant genes are involved in PVNH risk and that each individual variant is highly penetrant [γ (relative risk): >4k] (S1 Fig). However, we note that the wide confidence intervals on the parameter estimates suggest considerable uncertainty in these estimates and that much larger samples sizes will be needed to refine estimates of genetic architecture parameters in PVNH.

Since PVNH can have highly variable clinical presentations, ranging from subclinical to severe, we hypothesized that in some cases the risk alleles may be inherited from clinically unaffected parents, as is well recognized to occur in *FLNA*-associated PVNH. To address this hypothesis, we performed an association test evaluating for enrichment of rare alleles across individual genes in cases compared to controls using a gene-based collapsing analysis [17]. We started with variant calls from exome sequence data generated from 196 PVNH cases (excluding the six samples where sequencing was from DNA extracted from a lymphoblastoid cell line) and 13,364 controls selected from other studies and non-enriched for neurodevelopmental, neuropsychiatric, or severe pediatric diseases. All ethnicities were included in the analysis and we ensured approximately equal proportions of ethnicities among cases and controls. After a relatedness check and principal component analysis (Methods, S2 Fig), a total of 196 cases and 13,151 controls remained for association analysis.

**Fig 1:**
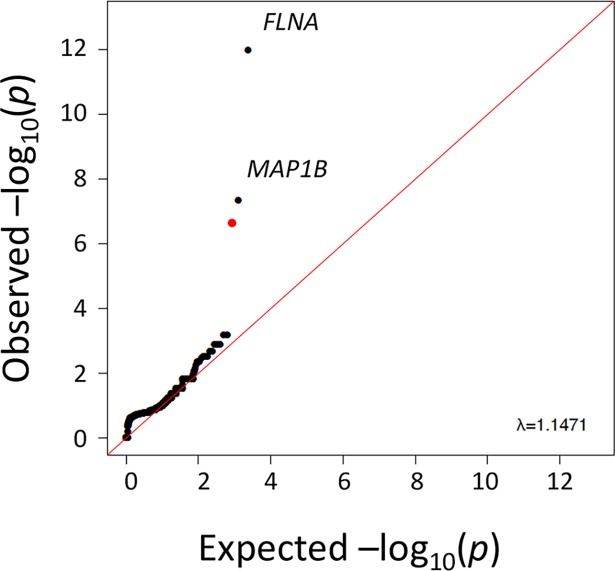
Quantile-quantile plot for gene-level association tests interrogating LoF variants. Black dots represent transformed p values against the expected transformed p values for genes with qualifying LoF variants. The red dot corresponds to the p value associated with *SON* however all four variants driving this signal were found to be false positives with Sanger sequencing. The red line indicates the expectation under the null model of no effect on risk.

To identify genes associated with PVNH under the case-control association analysis framework, we performed a genome-wide search for an over- or under-representation of “qualifying variants” in protein-coding genes in cases compared to controls—looking for genes where rare alleles confer risk or protection, respectively (Methods). Under a loss-of-function-only (LoF-only) model, where qualifying variants are required to be LoF variants (Methods), two genes (*FLNA* and *MAP1B*) showed enrichment of qualifying variants in PVNH patients with genome-wide significant p-values (Table 2, Fig 1). As a negative control, we also evaluated synonymous variants and found no enrichment of synonymous variants in cases or controls (S3 Fig). *SON* also had genome-wide significant enrichment of qualifying variants in cases, however this signal was driven by four *de novo* variants that were later found to be sequencing artifacts.

**Table 2:** Top associations from the gene-level case-control collapsing analyses.

LoF-only						
	Case carrier	%Case carrier	Control carrier	%Control carrier	Fisher’s exact test p-value	Rank of p-value among all genes tested
*FLNA*	7	3.57%	1	0.008%	1.1×10^−12^*	1/18405
*MAP1B*	4	2.04%	0	0%	4.5×10^−8^*	2/18405
LoF + missense (“probably damaging”)						
	Case carrier	%Case carrier	Control carrier	%Control carrier	Fisher’s exact test p-value	Rank of p-value among all genes tested
*FLNA*	12	6.12%	55	0.42%	2.1×10^−10^*	1/18405
*MAP1B*	4	2.04%	15	0.11%	1.5×10^−4^	2/18405

*Genome-wide significant (Methods).

**Fig 2:**
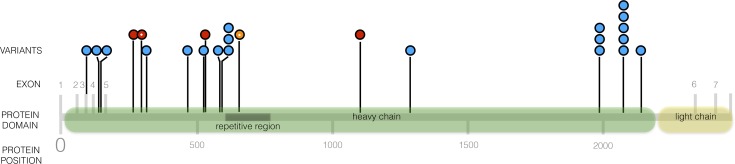
Distribution of *MAP1B* LOF alleles in PVNH cases (red dots), in individuals from ExAC and gnomAD databases (blue dots with number of alleles observed represented by number of dots running vertically at this site), and in the Deciphering Developmental Disorders case (orange dot). A Sanger confirmed *de novo* variant is indicated with a white dot in the circle.

Remarkably, *MAP1B*, a gene not previously known to be associated with PVNH, was the second most significant gene (following *FLNA*) owing to the presence of LoF qualifying variants in 4 of the 196 cases, and the absence of a LoF qualifying variant among 13,151 controls (Table 2). When we expanded the qualifying variants to include “probably damaging” missense variants (Methods), *FLNA* was the only significant association signal due to the additional contribution of missense qualifying variants, which have previously been demonstrated to be pathogenic in PVNH patients (Table 2). However, *MAP1B* became less significant because missense qualifying variants were found in 15 of the 13,151 controls and none of the cases (Table 2, S3 Fig). We further examined each of the four *MAP1B* LoF qualifying variants identified in the four PVNH cases. All four were heterozygous (Table 3), including one *de novo* and three inherited variants. None of the four cases had been resolved by a genetic diagnosis (e.g., *FLNA* or *NEDD4L* variants). All four variants are predicted to cause early premature truncation to the microtubule-associated protein 1B, which is 2,468 amino acids in length (Table 3, Fig 2). *MAP1B* is very intolerant to standing functional variation with an ExAC-based RVIS percentile of 2.27% and only 12 LOF variants (20 alleles) observed in the ExAC and gnomAD databases combined [29]. It is also a LoF-depleted gene achieving a ExAC-based FDR of 1.53x10^-11^ for preferential depletion of LoF variants [30] and a probability of being LoF intolerant (pLI) score of one [29, 31]. One additional *de novo* LOF variant in *MAP1B* was identified in a patient reported to have a range of phenotypes including an abnormality of the nervous system in the Deciphering Developmental Disorders Study (Fig 2, p.(Glu659Lysfs*22))[32], but MRI data that would allow for a diagnosis of PVNH was unavailable. All *MAP1B* variants were confirmed to be present with Sanger sequencing and the inheritance patterns were correctly inferred from the exome sequence data. *MAP1B* encodes a neuronal microtubule-associated protein that plays a key role in neurogenesis and neuronal migration through its effects on microtubule assembly and axon formation [33–35].

**Table 3:** *MAP1B* LoF qualifying variants identified in PVNH patients.

Variant ID (chr-position-ref-var, hg19)	Proband ID	Parental origin	Ethnicity (self-declared, inferred from genetic data)	Variant/reference depth (proband): (transmitting parent)	Annotation*
5-71490089-C-T	pvhnd29281lw1	*de novo*	American Indian—Alaska Native/European	63/70: NA	c.907C>T;p.(Arg303*)
5-71490776-C-T	pvhcw12701bvi1	inherited from father	European/European	38/31: 26/29	c.1594C>T;p.(Gln532*)
5-71492498-C-T	pvhnz9000cfc1	inherited from mother	Unknown/European	43/39: 35/26	c.3316C>T;p.(Arg1106*)
5-71489999-GC-G	pvhit1238Pbti1	inherited from father	European/European	44/50: 47/55	c.818delC;p.(Leu274Cysfs*4)

*Annotations are based on the canonical transcript NM_005909.3.

**Fig 3:**
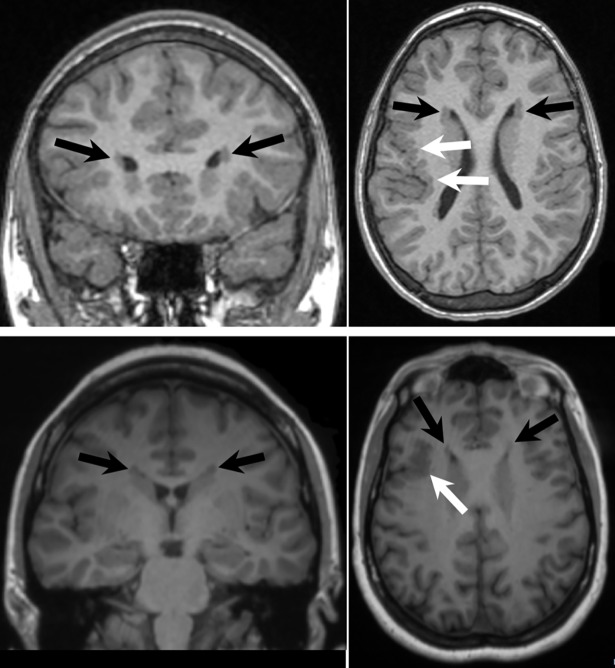
Brain MRI of subjects pvhnz9000cfc1 (top) and mother (bottom). Images are coronal T1-weighted (left column) and axial T1-weighted (right column). The images all show bilateral periventricular nodular grey matter heterotopia maximal in the frontal regions (black arrows). The axial images show over-folded cortex in the deep perisylvian/insular region on the right consistent with polymicrogyria (white arrows).

All of our patients with a *MAP1B* variant have anterior PVNH, bilateral and symmetric in three, and two of the four have deep perisylvian / insular polymicrogyria (S2 Table, S4 Fig, Fig 3). The pattern of PVNH is distinctive in that the nodules are frontal-predominant. This compares to the typical *FLNA*-associated PVNH in which the nodules are maximal along the bodies of the lateral ventricles, and the posterior or infrasylvian form of PVNH in which the nodules are maximal along the atria and temporal horns [36]. Seizures, cognitive impairment, and other dysmorphic features were variable across the four patients. Only one of the three transmitting parents (mother of pvhnz9000cfc1) reported having possible neurological symptoms and was available for additional clinical evaluation. Interestingly, both the mother and child in this family had similar neuroimaging findings, consisting of bilateral anterior PVNH and deep perisylvian and insular polymicrogyria (Fig 3), although the extent of the brain malformation was much milder in the mother. This distinctive phenotype in both mother and child with the same *MAP1B* variant, and the rareness of the phenotype in patients with PVNH [37], further implicates *MAP1B* in PVNH. The other two parents from whom probands inherited variants in *MAP1B* did not report neurological symptoms and had not undergone neuroimaging.

We next evaluated if we could detect additional association signals by looking across sets of genes comprising a pathway. To do so, we first removed the two marginally significant genes (*FLNA* and *MAP1B*) as well as the gene generating an artifactual signal (*SON*) from these analyses. We interrogated the 10,705 pathways defined in the GSEA Hallmark and C2 Gene Sets, and Gene Ontology defined gene sets [38–40]. To assess whether there was any evidence of residual non-null signal across genes in each pathway we used a higher criticism approach (Methods) [41] that is especially sensitive to detecting low-level signals across a series of genes. No pathway was significantly associated with PVNH status after removing the gene-level signals driven by *FLNA* and *MAP1B* variants. These analyses suggest that if additional association signal is present in the dataset, we are either underpowered to detect it or the relevant pathways are not captured amongst the gene sets evaluated.

Two biallelic risk models, one including LoF only and one LoF and “probably damaging” missense variants, were also evaluated considering a combination of both recessive or compound heterozygous qualifying variants using a case-control collapsing approach. No statistically significant association signals were detected and no gene had more than two qualifying genotypes in PVNH cases. No disease-causing biallelic genotypes were detected in the four known recessive PNVH genes *ARFGEF2*, *FAT4*, *DCHS1*, *INTS8*, *MCPH1* or *EML1*. Newly recessive, compound heterozygous, and newly hemizygous genotypes identified in the PVNH trios are provided in S3 and S4 Tables.

**Fig 4:**
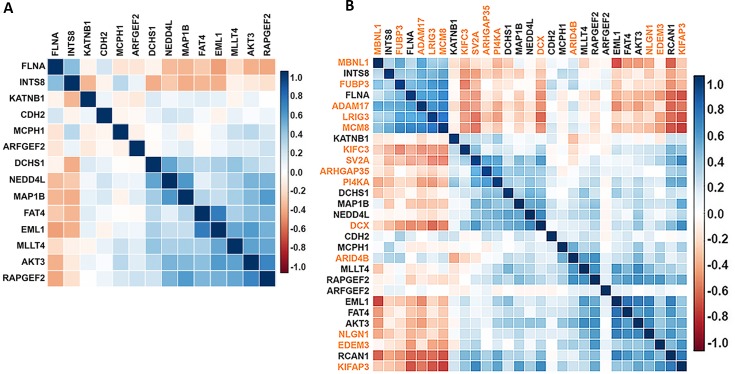
Ordered correlation matrices for the PVNH query and the fourteen loci significantly co-expressing within this node. Pairwise Pearson’s correlation represented as a matrix between (a) pairs of the 14 genes within the PVNH gene set (Methods) and (b) the human PVNH query plus the 14 genes whose co-regulatory patterns significantly exceed the eFDR in both the Kang and Miller transcriptomic datasets. Genes are ordered according to hierarchical clustering, with the most positive (+1) and negative (-1) co-regulatory interactions represented as blue and red squares, respectively.

Recently, it has been shown that epileptic encephalopathy genes tend to be co-expressed in the brain during development, and that, for this epilepsy phenotype, identifying genes harboring a single *de novo* mutation in a trio-based study that are transcriptionally co-regulated with known disease genes can effectively pinpoint genes that will be associated with the phenotype in larger cohorts [19, 20]. Both the hot-zone and the architecture analyses suggest that there are additional pathogenic *de novo* variants beyond the *de novo* variant in *MAP1B* and *NEDD4L* and those found in *FLNA*. In order to nominate candidate PVNH genes amongst the set with a *de novo* mutation in one of the 202 PVNH cases evaluated in this study, we evaluated human brain development developmental co-expression patterns of *de novo* mutation carrying genes with known PVNH genes. Since the prioritization approach will be most effective in cases where known genes tend to be co-expressed, we first evaluated if a set of human and rodent PVNH genes [(n = 14) Methods], exhibit greater co-expression than random sets of 14 genes during brain development (Methods). We showed that, among 1,000 randomly selected 14-gene sets, PVNH genes, including *MAP1B*, tend to have higher correlation coefficients when evaluating all possible two gene correlations within the 14 PVNH gene set (S5 Fig)[42–44]. We next evaluated the prioritization procedure (Methods) using a leave-one-out approach (S2 Text) where a known PVNH gene is removed from the list and evaluated to see if it would be subsequently prioritized. We found this approach was able to successfully reprioritize more PVNH genes than expected by chance (S5 Table). Based on these analyses, we compiled a list of all genes harboring at least one *de novo* variant predicted to alter the encoded protein and excluded those occurring in any of the 14 known PVNH loci to identify the genes that are co-expressed with known PVNH genes (S2 Text). Using this approach, 14/107 candidate genes exceeded the empirical significance cut-off in both transcriptomic datasets analyzed. These genes included *LRIG3*, *MBNL1*, *ARID4B*, *NLGN1*, *KIFC3*, *SV2A*, *ADAM17*, *KIFAP3*, *FUBP3*, *ARHGAP35*, *PI4KA*, *MCM8*, *EDEM3*, and *DCX* (S6 Table). Co-expression heatmaps showing the modules of co-regulation of known and prioritized PVNH genes are provided in Fig 4. The patterns of co-expression of these candidate PVNH genes with human PVNH genes across development are also provided in S6 Fig. These 14 genes harboring *de novo* variants in our 202 trios that co-express with known PVNH genes should be considered candidate PVNH genes, particularly those with hot-zone variants (*MBNL1*, *ARID4B*, *NLGN1*, *ARHGAP35*, *EDEM3* and *DCX*).

## Discussion

The PVNH cohort of 202 cases analyzed in this study was assembled with the goal of identifying novel variants and genes for this disorder. In this cohort we sought to identify disease-causing *de novo* variants considering the standard *de novo* variant paradigm in sporadic disease, and also rare inherited risk alleles since PVNH can exist in patients with subtle clinical or purely radiological presentations.

Using a trio approach, no gene in this study showed a genome-wide significant enrichment of *de novo* variants, other than *FLNA*, an already established PVNH gene. Despite this, results from our hot-zone analyses estimate that approximately 15 patients (7.4% of the cohort) harbor a non-*FLNA de novo* pathogenic variant, despite our inability in this small cohort to pinpoint these specific variants. This is further supported by the highly significant enrichment of nonsynonymous *de novo* variants in intolerant genes in the architecture analysis.

While we cannot pinpoint the exact pathogenic *de novo* variants outside of those in known PVNH genes, we suspect that a number of genes harbor pathogenic variants based either on meeting the hot-zone criteria or showing evidence of co-expression in the brain with known PVNH genes during the critical developmental time period. In total we identified 35 variants meeting the hot-zone criteria in 29 genes (S1 Table). Among these hot-zone *de novo* variants was one located in *MAP1B*, a gene implicated in this study through the gene-level collapsing analysis. *CHD5* was also found to harbor one hot-zone *de novo* variant, along with one synonymous *de novo* variant found in 0.004% of controls (S1 Table) and one missense variant predicted to be benign by Polyphen-2. Despite observing three *de novo* variants in *CHD5*, this pattern could occur by chance accounting for the mutability, predicted impact of the variants, and the size of the gene in a cohort of this size. However, this is an interesting candidate gene given what is known about the biological role of this gene. *CHD5* encodes the chromatin-remodeling protein chromodomain helicase DNA binding protein 5, which binds DNA and regulates transcription[45, 46]. *CHD5* expression is restricted to the brain where it activates genes promoting neuron terminal differentiation. Acute knockdown of *CHD5* within the developing mouse cortex, via *in utero* electroporation, impairs radial migration and causes a failure of cells to reach the cortical plate[47]. Additional studies will be needed to confirm or disprove this candidate association.

Further complementing the hot-zone analyses, we also used brain-specific human transcriptomic resources to nominate candidate genes based on their co-regulatory expression patterns with known PVNH genes. Interestingly, known disease-causing PVNH genes form distinct patterns of co-expression with loci that produce similar phenotypes, suggesting the co-expression networks outlined here are supportive of a common pathway. For example, the expression patterns of *FLNA* and *INTS8* are highly correlated across development (Fig 4). Pathogenic variants in *FLNA* produce a phenotype of symmetrically distributed heterotopia predominantly lining the anterior horns and ventricular bodies of the lateral ventricles. Hypoplasia of the cerebellar vermis and posterior fossa cysts are common accompaniments [12]. A very similar clinical phenotype is produced by variants in *INTS8* [10]. Although the genes significantly co-expressing with the query PVNH set should be viewed only as candidate PVNH loci several are also hot-zone variants, further reinforcing their potential role in PVNH.

Using a gene-level collapsing analysis to assess enrichment for both inherited and *de novo* alleles in PVNH cases, we identified a significant enrichment of loss-of-function variants in *MAP1B* in cases compared to controls, allowing us to clearly implicate this gene in PVNH risk. Interestingly, three of the four *MAP1B* variants driving the association signal were found to be transmitted from a unaffected parent, explaining why it was not identified in the initial trio analysis. None of the three inherited *MAP1B* variants showed evidence of mosaicism based on the number of reads supporting the variant compared to reference (Table 3).

*MAP1B*, encoding microtubule associated protein 1B, is involved in regulating both microtubule and actin dynamics. Specifically, *MAP1B* is encoded as a single peptide with one cleavage site located near the C-terminus. The subsequent cleavage of *MAP1B* induces the production of a heavy and light chain that can both interact with microtubules [48]. Neurons lacking *MAP1B* have reduced Rac1 and Cdc42 activity, with a concomitant increase in RhoA [49]. Changes in neurite extension and synapse development have also been associated with *MAP1B* modulation [50]. Although *MAP1B* is most commonly associated with roles in postmitotic neurons, a recent study in zebrafish indicates a role for Map1b earlier in neural convergence and neural tube development [51]. This role may also be relevant for the formation of PVNH where early functions in epithelial adherens junction formation have also been implicated [52–54]. *MAP1B* transcripts are predominantly detected in the early stages of cortical development where they are also negatively regulated by the Fragile X mental retardation protein (FMRP), an important cellular process contributing to various neurodevelopmental diseases [55, 56]. Interestingly, PVNH has also been reported in patients with Fragile X syndrome due to marked expansion and instability of the CGG trinucleotide repeat within the *FMR1* gene [57].

In addition to implicating a novel gene in PVNH, one of the most interesting aspects of this work is the idea that sporadic disease may, in some cases, be due to deleterious variants that arise in the germline in earlier antecedents to the proband yet for some reason fail to give rise to a phenotype in these individuals. While non-penetrance is always a consideration in genetic risk, the unique component here is that the *MAP1B* variants identified in this study are very rare (absent from at least 150K samples encompassing all public and internal databases) and loss-of-function variants are virtually absent from the population as well, a bioinformatics signature that is consistent with disease-causing *de novo* variants. This suggests that LOF variants in *MAP1B* would be likely to have occurred in very recent generations. Such a pattern has been documented for rare deleterious copy number variants where high-risk deletions or duplications have been shown to be transmitted from a clinically unaffected first or second degree relative, but this is only very rarely reported for sporadic diseases caused by point or small insertion-deletion variants [58, 59]. In fact this very phenomenon has been described probabilistically and shown not only to be possible but likely, depending on the disease penetrance and reproductive fitness conferred by the variants in question [60]. Interestingly, Kosmicki et al. recently reported over-transmission of LoF variants in LoF-depleted genes in a large cohort of sporadic autism spectrum disorder, a finding consistent with some transmitted alleles conferring risk [59]. This expansion of the *de novo* paradigm in PVNH may be in part due to the syndrome’s potential to result in sub-clinical phenotypes, but it may also represent the tip of the iceberg for a much more widespread effect in sporadic disease risk that has largely not been considered in most trio-based studies performed to date.

## Methods

### Ethics statement

The study was performed according to the standards of the ethics committees and the institutional review boards at each institute. Columbia University Medical Center’s Institutional Review Board centrally reviewed the approvals from each site under protocol number AAAP0052.

### Patient ascertainment and phenotyping

PVNH patients were assembled from multiple patient collections sites, including: (1) multiple sites encompassing the Epilepsy Phenome/Genome Project (EPGP, www.epgp.org) Cohort (n = 70), University of Florence’s Anna Meyer Children’s Hospital (n = 22), Boston Children’s Hospital (n = 24), University of Washington (n = 12), University of Otago (n = 65), and the Royal Children’s Hospital Melbourne (n = 10). All samples had presumed sporadic disease based on patient and family interview, and all except for a subset in the EPGP cohort were prescreened either clinically or in the research setting for disease-causing *FLNA* variants. MRIs were reviewed for the EPGP cohort as previously described [14] and for the additional cohorts by the enrolling sites. Patients enrolled into the EPGP cohort were excluded if an *FLNA* variant had been previously identified although not all patients underwent genetic testing for *FLNA* variants. EPGP inclusion criteria included the presence of epilepsy, whereas patients in other cohorts did not necessarily have epilepsy.

For comparison, 13,198 individuals who were sequenced as part of other genetic studies in the IGM were used as controls in this study. Approximately 6900 were neuropsychiatrically normal to our knowledge, and the remaining subjects had conditions where there are no known co-morbidities with epilepsy or brain malformations (S7 Table).

### Exome sequencing

Exome sequencing was performed on DNA from 202 probands and their parents at the Institute for Genomic Medicine (IGM, Columbia University), the Dunedin School of Medicine (University of Otago, New Zealand), and the Institute for Applied Genomics (Udine, Italy) (S8 Table). All externally-generated raw data were transferred to the IGM, where a combined analysis was performed using the same alignment and variant calling pipeline. The alignment and variant calling details have been previously reported [21]. Six of the 202 trios studied had one or more samples from the family exome sequenced from DNA extracted from a lymphoblastoid cell line (LCL); all others were sequenced from primary DNA sources (S8 Table).

### *De novo* variant calling

Candidate *de novo* variants were jointly called with the GATK Unified Genotyper for all family members in a trio as described previously [21]. Variants not located in the exonic region or splice sites (2-basepairs flanking an exon) defined by the Consensus Coding Sequence (CCDS, release 14, GRCh37.p13) were excluded. On average ~20 *de novo* single nucleotide variants were called per individual using this permissive calling approach. To remove the false positives from the dataset, we used Sanger sequencing validation results of a subset of *de novo* single variant calls from a subset of the PVNH trio cohort and from 403 individuals analyzed as part of other trio sequencing studies performed in the IGM to fit a machine-learning model using variant-level, individual-level, and genomic features to predict true positives (S3 Text, S7 Table). Trios sequenced from DNA from LCLs were excluded from this analysis. A fraction of the trios sequenced at another site were also excluded from this model because of insurmountable batch effect issues that confounded the model predictions. The resulting data set was comprised of 401 Sanger validated and 317 Sanger refuted (including inherited variants and variants not confirmed in proband) from 535 (132 PVNH and 403 from other studies) trios. Cross-validation was used to estimate the model’s accuracy, which was found to have high sensitivity (98%) and specificity (93%) (S3 Text). Confident in the model’s ability to predict true and false *de novo* calls, we then applied the model to the 9,172 de novo variant calls where Sanger sequencing was not performed. For each variant evaluated, the model assigned a probability score reflecting how likely the call is a true *de novo* variant. A score approaching one had a high probability of being a true *de novo* variant, and a score approaching zero had a low probability of being a true *de novo* variant (S7 Fig). We then set a threshold probability score for declaring a true *de novo* variant using the expected number of autosomal synonymous *de novo* variants per trio of 0.303 which translated to an expectation of 162 autosomal synonymous *de novo* variants across the cohort of 535 trios. The expected per trio rate of autosomal *de novo* synonymous variants was calculated by taking sum of the estimated trinucleotide mutation rate [27, 61, 62] across all possible substitutions in the autosomal protein-coding sequence that would not result in a change in the protein-coding sequence and multiplying by two to account for the two chromosomes. The threshold probability score for declaring a *de novo* variant true was set to 0.978, which allowed for 162 total *de novo* variants to be accepted as true either via direct Sanger confirmation or by having the highest probability score in the model.

Since *de novo* variants called in LCL trios, indel *de novo* variants, and those from trios beset by confounding batch effects were not analyzed in the model approach, we Sanger sequenced the majority of *de novo* calls that we felt may contribute to PVNH, including those that passed quality control filters and those that were absent in IGM controls and the ExAC and EVS databases. Quality control filters included: single nucleotide variant calls were excluded if with QD < 2.0, MQ < 40.0, FS > 60.0, HS > 13.0, MQRS< -12.5, or RPRS < -8.0; indel variant calls were excluded if with QD < 2.0, RPRS < -20.0, or FS>200. More than 70% of this subset of calls were confirmed with Sanger sequencing.

A list of all Sanger confirmed and model predicted true *de novo* variants identified in the PVNH cohort (n = 219 *de novo* variants) are provided in S1 Table.

### Listing of newly recessive, compound heterozygous, and newly hemizygous genotypes in PVNH probands

We first identified all putatively protein-altering (missense, nonsense, or indels) residing in the protein-coding regions (CCDS, release 14, GRCh37.p13) newly recessive, compound heterozygous, and newly hemizygous genotypes in the PVNH probands by assessing the genotypes across the trios. Genotypes were excluded if they had a quality score (QUAL) <30 and a genotype quality (GQ) score of <20 in the proband. We also required a minimum coverage of 10-fold at a variant site to call a homozygous reference genotype. Newly recessive, compound heterozygous, and newly hemizygous genotypes were excluded if any contributing variant had a the minor allele frequency greater than 1% or if a recessive or hemizygous genotype was reported in the internal control cohort or any population in Exome Variant Server (EVS) and Exome Aggregate Consortium (ExAC release 0.3).

### Hot-zone analysis

Sanger confirmed and model-predicted true (see Methods) single nucleotide *de novo* substitutions found in PVNH cases and absent from in-house controls and the EVS and ExAC databases were first scored on their likelihood to alter the encoded protein. Trios with one or more samples sequenced from lymphoblastoid cell lines were excluded from this analysis. Synonymous and loss-of-function (nonsense, and splice acceptor/donor) variants were scored 0 and 1, respectively, and missense variants were scored using their Polyphen-2 score (HumVar). We next scored each *de novo* variant at the gene level using the gene-level residual variation intolerance percentile (RVIS, %RVIS_ExAC_0.05% (all populations), which ranks genes based on their tolerance to polymorphic functional genetic variation [63] on a scale from 0 to 1, with the higher the value the more tolerant the gene is to standing functional variation. For comparison, we also assessed *de novo* variants in previously published healthy control trios (n = 250 [64]) using the same annotations and filtering procedures used in this study. For each case and control sample with more than one *de novo* substitution meeting the aforementioned criteria, only the single most damaging *de novo* variant was used; i.e. the *de novo* variants with the shortest Euclidian distance from the most damaging coordinate [x = 1,y = 0] on a plot of the variant-level vector along the X-axis and the gene-level vector (RVIS percentile score) along the Y-axis. Individuals with no single nucleotide *de novo* substitutions did not contribute to this analysis. A two-tail Fisher’s exact was used to test whether the single most damaging *de novo* variants found in PVNH cases preferentially lie in the “hot-zone”, defined by a PolyPhen-2 score of ≥ 0.95 and RVIS ≤ 25^th^ percentile[63], compared to control trios.

### Gene-level collapsing analyses

Variants for analysis were restricted to the consensus coding sequence public transcripts (CCDS release 14) plus 2 base pair intronic extensions. Variants were further required to have: i) at least 10-fold coverage, ii) quality score (QUAL) of at least 30, iii) genotype quality (GQ) score of at least 20, iv) quality by depth (QD) score of at least 2, v) mapping quality (MQ) score of at least 40, vi) read position rank sum (RPRS) score greater than -3, vii) mapping quality rank sum (MQRS) score greater than -6, viii) indels were required to have a maximum Fisher’s strand bias (FS) of 200, ix) variants were screened according to VQSR tranche calculated using the known SNV sites from HapMap v3.3, dbSNP, and the Omni chip array from the 1000 Genomes Project to “PASS” SNVs were required to achieve a tranche of 99.9% for SNVs in genomes and exomes and 99% for indels in genomes, x) for heterozygous genotypes, the alternate allele ratio was required to be ≥25%. Finally, variants were excluded if they were among a predefined list of known sequencing artifacts or if they were marked by EVS (http://evs.gs.washington.edu/EVS/) or ExAC (http://exac.broadinstitute.org/about) as being problematic variants. Variants were annotated to Ensembl 73 using SnpEff. All variants meeting these criteria were eligible to be qualifying variants in the gene-based collapsing analyses. Additional filtering based on variant function or per inheritance models being tested were applied depending on the sub-analysis performed. We note that the case and control populations were pre-screened with both KING and PLINK to ensure only unrelated (up to second-degree) samples were used. Any exomes with gender discordance between clinically-reported and X:Y coverage ratios were removed, as were contaminated samples according to VerifyBamID. No PVNH cases were excluded with this filtering.

Before running gene-based collapsing analysis, we implemented both sample- and site-level pruning procedures to minimize the systemic bias in data that might lead to spurious association or reduced power to detect real association. The site-pruning procedure was performed as described previously[18]. Here, we described the sample-level pruning procedure including removing related individuals and population outliers identified in principal component analysis (PCA). To identify related individuals, we generated genotype data in PLINK format and then used KING to calculate pairwise kinship coefficients for all case and control subjects. No individual were found to be related greater than the kinship coefficient 0.1. Next we ran PCA using EIGENSTRAT with a LD-pruned (r^2^ threshold 0.2) list of single-nucleotide polymorphisms (SNPs) extracted from exome sequencing data.

Following cleaning of the dataset, we then assessed for enrichment or depletion of “qualifying variants” across cases and controls. A “qualifying variant” was defined by a set of criteria based on allele frequency and functional prediction of that variant, with the criteria designed to capture the characteristics of previously identified pathogenic variants causing PVNH. Specifically, in this study, a variant was determined to be qualifying in the dominant model if it 1) was absent in the Exome Variant Server (EVS) and Exome Aggregate Consortium (ExAC release 0.3), 2) had ≤4 copies of variant allele in the 196 cases plus 13,151 controls, and 3) was predicted to be loss-of-function (stop gained, frameshift, splice site acceptor, splice site donor, start lost, or exon deleted) or missense “probably damaging” by PolyPhen-2 (HumDiv).

In the bi-allelic model, that included compound heterozygous and recessive genotypes, a genotype was considered qualifying if 1) the variant site(s) was predicted to be loss-of-function (stop gained, frameshift, splice site acceptor, splice site donor, start lost, or exon deleted) or missense “probably damaging” by PolyPhen-2 (HumDiv), and 2) the variant(s) sites had a minor allele frequency of <0.001 in the Exome Variant Server (EVS), the Exome Aggregate Consortium (ExAC release 0.3), and across the case-control cohort.

For each gene, an indicator variable (1/0 states) was assigned to each individual based on the presence of at least one qualifying variant (dominant model) or genotype (bi-allelic model) in the gene (state 1) or no qualifying variant/genotype in that gene (state 0). We note that phasing of compound heterozygous variants was not taken into account in the collapsing analyses due to the fact that those data were not available for the control cohort. Two-tailed Fisher’s exact test was used to evaluate statistical significance of genic association. With 18,405 genes tested, we adopted the genome-wide significance level of p = 6.79×10^−7^ using Bonferroni correction correcting for all the genes in the genome and the four different models tested (0.05/18,405/4).

Quantile-quantile plots were generated using a permutation-based expectation. To achieve this, for each model (matrix) we randomly permuted the case and control labels of the original configuration: 196 cases and 13,151 controls and then recomputed the Fisher’s Exact test for all genes. This was repeated 1,000 times. For each of the 1,000 permutations we ordered the p-values and then took the mean of each rank-ordered estimate across the 1,000 permutations, i.e., the average 1st order statistic, the average 2nd order statistic, etc. Thus, these represent the empirical estimates of the expected ordered p-values (expected -log10(p-values)). This empirical-based expected p-value distribution no longer depends on an assumption that the p-values are uniformly distributed under the null.

### Pathway analyses

Analyses begin with marginal, gene-level p-values from a standard collapsing analysis. However, in order to optimize computational speed we use a standard chisquare test instead of Fisher’s Exact text. Since the ultimate null distribution was computed via permutation, we expect this change to have minimal impact. Analyses were performed across all 10,705 pathways defined in the GSEA Hallmark and C2 Gene Sets, and Gene Ontology defined gene sets [38–40]. The higher criticism (HC) test is obtained by maximizing a scaled difference between the observed distribution of gene-level p-values across the pathway and the distribution of p-values one would expect if all the genes were null [41]. We use permutation to compute this expectation. The HC test is not only sensitive to extreme p-values but also to more subtle shifts in the p-value distribution. We compute unweighted and weighted versions of the HC test, where the weighted version upweights genes that are especially important (node centrality or low genic intolerance) within the pathway. Since a given gene may be included in multiple pathways, the resulting pathway-level tests will correlated. Thus, in order to account for the large number of tests conducted while also taking this correlation into account, we use the permutation-based multiplicity adjustment procedure of Ge et al [65].

### PVNH gene set

We established a list of genes associated with PVNH and related phenotypes based on evidence from the rodent and human literature for use in the co-expression analyses. To compile this list we specifically included all genes for which mutations reproducibly produce subcortical or periventricular heterotopia in a substantial fraction of individuals, and excluded genes with only single reports of periventricular heterotopia in human. Nine genes have been previously reported to be human PVNH risk loci in more than one individual, including *FLNA*, *FAT4*, *DCHS1*, *ARFGEF2*, *C6orf70*, *AKT3*, *INTS8*, *MCPH1*, and *NEDD4L* [3–11]. Genes for which mutations are reported to cause a partial, diffuse, heterotopic malformation, specifically subcortical band heterotopia were excluded, however we did include genes associated with subcortical heterotopia often presenting with PVNH, including *GPSM2*[66], *EML1*[5] and *KATNB*1 [67]. In addition to human PVNH genes, we also included those genes in mice which when conditionally knocked-out induce impaired neuronal migration phenotypes analogous to, or closely resembling, subcortical or PVNH in humans, including *CTNNA1*, *RAPGEF2*, *RCAN1* and *MLLT4* [68–71]. We also include *MAP1B* based on the data presented in this report. *C6orf70*, *RCAN1* and *GPSM2* were not represented in all transcriptomic datasets analysed here and were therefore excluded. The PVNH genelist thus consisted of 14 genes.

### Brain transcriptomic datasets

We downloaded three publicly available transcriptomic datasets generated from post-mortem human brain. While all datasets contain only donors deemed to have normal brain development, they are very different with regards to number of sampled regions per donor, number of donors and ages of donors. For the Miller et al [42] dataset, on average 328 regions from 4 fetal brains were assayed. The Colantuoni et al dataset exclusively looked at the prefrontal cortex in 266 brains from fetuses, children and adults [44]. The Kang et al dataset similarly includes donors of all ages but sampled on average 57 regions per brain. Additional details of these datasets are provided in S9 Table. All three transcriptomic datasets contained post-mortem human brain expression data from the disease-relevant time periods (4–38 weeks post conception, S9 Table), however in some cases the data were limited. Although each dataset is built from various brain structures, for the purposes of these prioritizations this information was not used.

Using methods described previously [72], outlier samples were first removed, followed by normalization using the Removal Of Unwanted Variation (RUV) method (R package RUVcorr) that controls for systematic noise using negative control genes [73].

The three datasets used here were subsetted so that only the transcriptomic information within the disease relevant periods (4–38 weeks post conception) were used targeted for use in the candidate gene prioritization, however only two (Miller and Kang datasets) were found to have sufficient data to be useful in these analyses (S2 Text, S5 Table). Thus, co-expression analyses were limited to just the Miller and Kang transcriptomic datasets.

### Prioritization of genes harboring a *de novo* variant based on co-expression with known genes

In this study, we identified 107 genes harboring model predicted true or Sanger confirmed *de novo* variants, excluding *FLNA*, *NEDD4L* and *MAP1B*. To prioritize candidate PVNH gene based on co-expression with the 14 PVNH associated genes (see above), we first estimated the background correlation coefficient for any random 107 gene set whereby 20% of genes in this list would be prioritized. To do this we generated 1,000 sets each containing 107 randomly selected genes. In each random set, the pair-wise absolute weighted correlation between the expression of each of these random genes and all PVNH genes were calculated. The weighted correlation refers to correlations weighted by the inverse of the number of samples contributed by the respective donor. For any single gene in the 107 gene set, only the maximum correlation with a PVNH gene was retained, resulting in 107 correlation coefficients retained for each randomly selected gene set, and 18,200 total correlations across all 1,000 sets of genes. From this distribution of 18,200 maximum absolute correlations, the correlation coefficient threshold was that corresponding to the lowest value for the highest 20^th^ percentile was used as a threshold. We then calculated the correlation coefficient between genes harboring a *de novo* variant and the PVNH genes, and prioritized those with a correlation coefficient with any of the 14 PVNH genes greater than the threshold value.

## Supporting information

S1 Text *De novo* variant architecture analyses. (PDF)

S2 Text Evaluation of the co-expression prioritization method. (PDF)

S3 Text Predictive model for identifying true *de novo* variants. (PDF)

S4 Text Supporting information references. (PDF)

S5 Text Control sample acknowledgments. (PDF)

S6 Text Specific contributions of authors, members of the Epi4K Consortium and Epilepsy Phenome/Genome Projects, and additional participants involved in this work. (PDF)

S1 Table De novo variants identified in PVNH cases. (XLSX)

S2 Table Phenotypes of patients and transmitting parents where available. (PDF)

S3 Table Homozygous and hemizygous variants identified in PVNH cases. (XLSX)

S4 Table Compound heterozygous mutations identified in PVNH cases. (XLSX)

S5 Table PVNH genes prioritized in the leave one out analysis across the three datasets. (PDF)

S6 Table Candidate genes prioritized based on co-regulation with known PNVH genes during the key developmental period (4–38 pcw). (XLSX)

S7 Table Control cohort composition. (PDF)

S8 Table PVNH cohort summary. (XLSX)

S9 Table Overview of transcriptomic datasets used for co-expression analyses. (PDF)

S10 Table Features used in the predictive model and their relative influence in the *de novo* variant confirmation model. (PDF)

S1 Fig Likelihood surface representing the genetic architecture of periventricular nodular heterotopia. (PDF)

S2 Fig Eigenvectors of cases (red dots) and controls (blue dots) across top three principal components from eigenstrat analyses. (PDF)

S3 Fig Quantile-quantile plot for gene-level association tests interrogating (A) LoF and “probably damaging” (Polyphen-2) missense variants, and (B) synonymous variants.
(PDF)

S4 Fig Brain MRI of subjects with LoF MAP1B variants. The left image is coronal T1 inversion recovery (pvhit1238Pbti1), the middle image is coronal T2-weighted (pvhnd29281lw1) and the right image is axial T2 weighted (pvhcw12701bvi1). All images show periventricular nodular grey matter heterotopia maximal in the frontal regions (arrows). No polymicrogyria was seen in subjects pvhit1238Pbti1 or pvhnd29281lw1. There was possible right insular polymicrogyria in pvhcw12701bvi1, but the available images were not of sufficient quality to be conclusive. MRI image for fourth patient is provided in Fig 3.
(PDF)

S5 Fig Cumulative proportion of correlation coefficients for 14 genes implicated in PVNH compared to 1000 randomly selected sets of 14 genes. (PDF)

S6 Fig Correlation matrices for prioritized genes compared to each known human PVNH query gene individually and across different periods of brain development. Pairwise Pearson’s correlations between prioritized genes harboring de novo mutations based on co-expression profiles and the 8 human query genes through each time period analyzed and presented across each row. Patterns of positive (+1) and reciprocal (-1) co-regulatory interactions are represented as blue and red squares, respectively. Data was derived from the Miller and Kang transcriptomic datasets with the time points in which the data was derived being separated into specific periods as indicated in by the value below each point; 1, 4-8pcw; 2, 8-10pcw; 3, 10-13pcw; 4, 13-16pcw; 5, 16-19pcw; 6, 19-24pcw; 7, 24-38pcw; 8, birth-6M; 9, 6M-1Y; 10, 1-6Y; 11, 6-12Y; 12, 12-20Y; 13, 20-40Y; 14, 40-60Y; 15, 60Y+. pcw, weeks post conception; M, months after birth; Y, years after birth.
(PDF)

S7 Fig Histrogram of *de novo* variant confirmation probabilities. (PDF)
