# Supplementary material for: De novo and inherited private variants in MAP1B in periventricular nodular heterotopia
Source: PLoS Genet. 2018 May 8;14(5):e1007281. doi: 10.1371/journal.pgen.1007281 (PMC5965900; doi:10.1371/journal.pgen.1007281)
Supplement: S1 Text — (PDF) [file pgen.1007281.s001.pdf]

S1 Text. *De novo* variant architecture analyses

We conducted a likelihood analysis of parameters describing the genetic architecture of periventricular nodular heterotopia, including the relative risk (γ) and proportion of the exome related to periventricular nodular heterotopia (η). As there were substantial differences in sequenced regions across the different exome sequencing methods used in this study, we adapted our previously proposed likelihood model to incorporate trio-specific mutation rates that take into account the "callable real-estate" and observed *de novo* variants. This likelihood can be written as

$$L(\gamma,\eta) = \prod_{i} \left\{ \frac{\frac{\lambda_i}{x_i!} e^{-\lambda_i} \left[\gamma + (1-\gamma)(1-C\eta)^{x_i}\right]}{\gamma + (1-\gamma) e^{-\lambda_i C\eta}} \right\}$$

where x_i and λ_i are the *de novo* variant counts and mutation rate, respectively, for the ith trio. As in previous analyses, C is assumed to be a known constant, but since the d*e novo* architecture is here restricted to nonsynonymous variation and splice sites are not considered, the value is taken to be 0.335 (estimate 30% of missence variants are deleterious and 3.5% are nonsense). Point estimates were obtained by optimizing equation 1 and likelihood ratio tests are computed by comparing the log-likelihood at this optimum to the value obtained under the null hypothesis.
