# Supplementary material for: De novo and inherited private variants in MAP1B in periventricular nodular heterotopia
Source: PLoS Genet. 2018 May 8;14(5):e1007281. doi: 10.1371/journal.pgen.1007281 (PMC5965900; doi:10.1371/journal.pgen.1007281)
Supplement: S2 Text — (PDF) [file pgen.1007281.s002.pdf]

## S2 Text. Evaluation of the co-expression prioritization method

Three transcriptomic databases were used in these analyses and are summarized below in S9 Table. We evaluated the performance of the co-expression prioritization method used in this study in two ways. First, we tested whether the 14 known PVNH genes do in fact appear to show evidence of being co-expressed compared to random similarly sized gene sets. To do this we first, generating 1000 sets of 14 genes randomly selected from the genome. We then calculated the 196 correlation coefficients corresponding to each possible two-gene comparisons across each set of 14 genes. We then plotted the cumulative proportion of correlation coefficients across binned values from 0-1. In the Miller transcriptomic dataset, the PVNH genes correlation values tended to be higher than random gene sets, however, for both the Kang and Colantuoni datasets the PVNH gene behaved similarly to the randomly generated gene lists (S6 Fig.).

We next assessed the ability of each transcriptomic dataset to predict the established PVNH gene set using a leave-one-out cross validation with the set of known PVNH genes. The leave-one-out cross-validation was modelled on the approach described by Aerts et al [1]. In this approach one gene is deleted from the known set of genes and termed the "defector" gene. The ability to prioritize this gene in a list of 99 other candidates, made up of random genes not known to be associated, determines the accuracy. Since the prioritization procedure prioritizes the top 20% of all genes with the highest two gene correlation coefficients with the disease gene set, we expect that at least 20% of PVNH genes should be prioritized in any single transcriptomic dataset. The Miller and Kang datasets performed well, prioritizing 9/14 genes (64%) and 8/14 (57%), respectively (S5 Table). Strikingly the Colantuoni dataset failed to prioritize any of the genes in the PVNH set. Given the failure of the Colantuoni dataset to prioritize any PVNH genes it was excluded from being used in the prioritization procedure to predict genes with *de novo* variants identified in this study.
