# Supplementary material for: De novo and inherited private variants in MAP1B in periventricular nodular heterotopia
Source: PLoS Genet. 2018 May 8;14(5):e1007281. doi: 10.1371/journal.pgen.1007281 (PMC5965900; doi:10.1371/journal.pgen.1007281)
Supplement: S3 Text — (PDF) [file pgen.1007281.s003.pdf]

S3 Text. Predictive model for identifying true *de novo* variants

We used a gradient-boosted machine learning logistic regression model as implemented in the R package gbm (https://cran.r-project.org/web/packages/gbm/gbm.pdf). The gradient-boosting algorithm produces a prediction model in the form of an ensemble of weak tree-based models. Trees are added to the ensemble based on their ability to reduce residual loss (logistic loss function in our case). Using this approach, we developed a predictive model using 718 variants confirmed by Sanger sequencing (401 confirmed *de novo*; 317 found not to be *de novo*) and features presented in S10 Table. We used 10-fold cross validation to identify the total number of trees (26k) and the maximum interaction depth (2) that minimizes prediction error (sensitivity, 98%; specificity, 93%). S10 Table also lists the relative importance of each feature within the model [2]. We then used this model to predict whether 9,172 potential *de novo* mutations that were not Sanger sequenced would be likely to confirmed if Sanger sequencing was performed. Based on the variants' features (S10 Table) the model gives an estimate of the probability that the potential *de novo* would be confirmed. We calibrated a threshold for this probability, by establishing the rate of potential synonymous *de novos* that would be classified as confirmed at a given threshold. We then use the threshold where the number of predicted true synonymous *de novo* mutations agrees with the expected number of synonymous mutations given the mutation rate of synonymous callable sequence. This threshold was then applied to potential nonsynonymous *de novo* and those predicted to confirm were then used the architecture analyses described below.

Each model prediction was associated with a probability score ranging from zero (low confidence) to one (high confidence). A histrogram showing the distribution of scores is provided in S7 Fig.
