# Supplementary material for: De novo and inherited private variants in MAP1B in periventricular nodular heterotopia
Source: PLoS Genet. 2018 May 8;14(5):e1007281. doi: 10.1371/journal.pgen.1007281 (PMC5965900; doi:10.1371/journal.pgen.1007281)
Supplement: S4 Text — (PDF) [file pgen.1007281.s004.pdf]

S4 Text. Supporting Information References

1. Aerts S, van Helden J, Sand O, Hassan BA. Fine-tuning enhancer models to predict transcriptional targets across multiple genomes. PLoS One. 2007;2(11):e1115. doi: 10.1371/journal.pone.0001115. PubMed PMID: 17973026; PubMed Central PMCID: PMCPMC2047340.
2. Friedman JH. Greedy function approximation: A gradient boosting machine. Ann Stat. 2001;29(5):1189-232. doi: DOI 10.1214/aos/1013203451. PubMed PMID: WOS:000173361700001.
3. Kang HJ, Kawasawa YI, Cheng F, Zhu Y, Xu X, Li M, et al. Spatio-temporal transcriptome of the human brain. Nature. 2011;478(7370):483-9. doi: 10.1038/nature10523. PubMed PMID: 22031440; PubMed Central PMCID: PMCPMC3566780.
4. Miller JA, Ding SL, Sunkin SM, Smith KA, Ng L, Szafer A, et al. Transcriptional landscape of the prenatal human brain. Nature. 2014;508(7495):199-206. doi: 10.1038/nature13185. PubMed PMID: 24695229; PubMed Central PMCID: PMCPMC4105188.
5. Colantuoni C, Lipska BK, Ye T, Hyde TM, Tao R, Leek JT, et al. Temporal dynamics and genetic control of transcription in the human prefrontal cortex. Nature. 2011;478(7370):519-23. doi: 10.1038/nature10524. PubMed PMID: 22031444; PubMed Central PMCID: PMCPMC3510670.
