# Supplementary material for: De novo and inherited private variants in MAP1B in periventricular nodular heterotopia
Source: PLoS Genet. 2018 May 8;14(5):e1007281. doi: 10.1371/journal.pgen.1007281 (PMC5965900; doi:10.1371/journal.pgen.1007281)
Supplement: S5 Text — (PDF) [file pgen.1007281.s005.pdf]

S5 Text. Control sample acknowledgements

We would like to acknowledge the following individuals or groups for the contributions of control samples: K. Welsh-Bomer; C. Hulette; J. Burke; D. Valle; J. Hoover-Fong; N. Sobriera; A. Poduri; R. Buckley; D. Murdock; R. Ottman; S. Delaney; E. Holtzman; G. Cavalleri; N. Delanty; S. Hirose; V. Shashi; M. Carrington; C. Woods; Croasdaile Village; K. Schmader; S. McDonald; M. Yanamadala; H. White; P. Lugar; G. Nestadt; S. Schuman; E. Nading; S. Palmer; E. Pras; D. Lancet; Z. Farfel; T. Young; K. Whisenhunt; C. Chen; J. Milner; C. Moylan; A. M. Diehl; M. Abdelmalek; D. Daskalakis; M. Winn, R. Gbadegesin; M. Hauser; A. Holden, E. Behr; H.B. Simpson; M. Walker; M. Sum; M. Connors; L. Morris; V. Shashi, K. Schoch; M. Harms; T. Miller; A. Pestronk; R. Bedlack; E. Cirulli; K, Grace; R. Brown; N. Shneider; S. Gibson; J. Ravits; A. Gitler; J. Glass; F. Baas; S. Appel; E. Simpson; G. Rouleau; the ALS Sequencing Consortium; the Epi4K Consortium and Epilepsy Phenome/Genome Project; the National Institute of Allergy and Infectious Diseases Center for HIV/AIDS Vaccine Immunology (CHAVI); the members of the Center for HIV-AIDS Vaccine Immunology; Duke University Health System Nonalcoholic Fatty Liver Disease Research Database and Specimen Repository; Genomic Translation for ALS Care (GTAC) study; the Epi4K Consortium and Epilepsy Phenome/Genome Project.

The collection of control samples and data was funded in part by: Biogen, Inc.; Gilead Sciences, Inc.; UCB; Bryan ADRC NIA P30AG028377; B57 SAIC-Fredrick Inc M11-074; National Institute of Neurological Disorders and Stroke (RC2NS070344; RC2MH089915; U01NS077303; U01NS053998, U54NS078059, P01HD080642); National Human Genome Research Institute (Yale Mendelian Genomics Center - UM1HG006504, U01HG007672); National Institute of Mental Health (K01MH098126, R01MH097971, R01MH099216, RC2MH089915); National Institute of Diabetes and Digestive and Kidney Diseases (R01DK080099); National Institute of Allergy and Infectious Diseases (Division of Intramural Research, 1R56AI098588-01A1); National Institute of Allergy and Infectious Diseases Center for HIV/AIDS Vaccine Immunology and Immunogen Discovery (UM1AI100645, U19AI067854); National Center for Advancing Translational Sciences (UL1TR000040); Eunice Kennedy Shriver National Institute of Child Health and Human Development(R01HD048805); the Ellison Medical Foundation New Scholar award AG-NS-0441-08; the Duke Chancellor's Discovery Program Research Fund 2014; Neil Molberger Brain Research Fund; Endocrine Fellows Foundation Grant; Bill and Melinda Gates Foundation; The Murdock Study Community Registry and Biorepository; The Stanley Institute for Cognitive Genomics at Cold Spring Harbor Laboratory; the Duke Genome Sequencing Clinic; New York-Presbyterian Hospital; Columbia University College Physicians and Surgeons; Columbia University Medical Center; The J. Willard and Alice S. Marriott Foundation; The Muscular Dystrophy Association; The Nicholas Nunno Foundation; The JDM Fund for Mitochondrial Research; The Arturo Estopinan TK2 Research Fund; and The Endocrine Fellows Foundation; Helaine B Allen and Emily Allen Wolff.

Data collection and sharing for the WHICAP project (used as controls in this analysis) was supported by the Washington Heights-Inwood Columbia Aging Project (WHICAP, PO1AG07232, R01AG037212, RF1AG054023) funded by the National Institute on Aging (NIA) and by the National Center for Advancing Translational Sciences, National Institutes of Health, through Grant Number UL1TR001873. This manuscript has been reviewed by WHICAP investigators for scientific content and consistency of data interpretation with previous WHICAP Study publications. We acknowledge the WHICAP study participants and the WHICAP research and support staff for their contributions to this study.
