# Supplementary material for: De novo and inherited private variants in MAP1B in periventricular nodular heterotopia
Source: PLoS Genet. 2018 May 8;14(5):e1007281. doi: 10.1371/journal.pgen.1007281 (PMC5965900; doi:10.1371/journal.pgen.1007281)
Supplement: S6 Text — (PDF) [file pgen.1007281.s006.pdf]

S6 Text. Specific contributions of authors, members of the Epi4K Consortium and Epilepsy Phenome Genome Projects, and additional participants involved in this work.

ACO, AGM, AP, ASA, CAW, DBG, DD, DHL, EEE, EHS, ELH, HCM, IES, JC, MPE, MRJ, ND, PCO, RG, RKU, RL, RO, SFB, StPet, SlPet, SPR, TG, TJO, and WBD designed arms of the study that were ultimately merged into one study. All aspects of the work were overseen by ASA, DBG, DHL, ELH, and SFB, and supported administratively by CF. AB, AEF, BAK, DA, DC, DD, DF, DP, EA, EG, EHS, EK, EPGV, EPK, FA, FD, GDC, GKVA, HK, IES, IS, JB, JJS, JMPao, JMPar, JS, KH, MCS, MHC, MRS, NF, OD, PCr, PVM, PWW, RAS, Rku, RKn, RKS, SG, SH, SJ, SS, RL, RO, SPR, and WBD were involved with patient sample collection. The phenotyping of study participants was performed by AB, ACO, AEF, AP, BAK, CF, CAW, DA, DC, DD, DF, DP, EA, EG, EHS, EK, EPGV, EPK, FA, FD, GDC, GKVA, GM, HK, IES, IS, JB, JC, JJS, JMPao, JMPar, JS, KH, MCS, MHC, MRS, NF, OD, PCr, PVM, PWW, RAS, RG, Rku, RKn, RKS, RL, RO, SG, SH, SJ, SPR, SS, and WBD. Samples were processed for genetic analysis by CMa, CMe, and PL. Sequence data was generated by CMa and CMe. The genetic analysis strategy was developed by ASA, DBG, ELH, and SP, and the analysis software was developed and programmed by ASA, BC, SlPet and ZR. Bioinformatic processing of data was performed by BC, DM, JSB, NGG, NS, SK, and ZR. Analysis and interpretation of the genetic data was led by ASA, DBG, ELH, XZ and ACO with input from DHL, CAW, RG, RL, SPR, SFB, SlPet, and WBD. Brain transcriptomic data analysis and interpretation was performed by ACO, MB, SF. Pathway analyses were performed by ASA and MZ. The manuscript was written by ACO, AP, ASA, DBG, DHL, ELH, SF, SPR, SFB, and XZ. The collective activities of the Consortia were overseen by DHL (EPGP) and DBG, DHL, and SFB (Epi4K).
