# Supplementary material for: De novo and inherited private variants in MAP1B in periventricular nodular heterotopia
Source: PLoS Genet. 2018 May 8;14(5):e1007281. doi: 10.1371/journal.pgen.1007281 (PMC5965900; doi:10.1371/journal.pgen.1007281)
Supplement: S2 Table — (PDF) [file pgen.1007281.s008.pdf]

S2 Table. Phenotypes of patients and transmitting parents where available.

Subject ID	pvhit1238Pbti1	pvhnd29281lw1	pvhcw12701bvi1	pvhnz9000cfc1	mother of pvhnz9000cfc1
**variant**	5-71489999-GC-G	5-71490089-C-T	5-71490776-C-T	5-71492498-C-T	5-71492498-C-T
**inheritance**	inherited from father (no known MRI)	*de novo*	inherited from father (reported healthy, no known MRI)	inherited from mother with similar MRI and symptoms	unknown
**MRI-PVNH description**	bilateral PVNH: left frontal horn and right lateral ventricle, possible additional right posterior nodule	bilateral anterior predominant PVNH	bilateral anterior predominant PVNH	bilateral anterior predominant PVNH	bilateral anterior predominant PVNH
**MRI-PMG description**	none	none	possible R perisylvian PMG	deep perisylvian/insular PMG (R>L)	deep perisylvian/insular PMG (R>L), less prominent than her daughter's
**MRI-other features**		open opercula anteriorly, thin corpus callosum		thin, dysmorphic corpus callosum	thin, dysmorphic corpus callosum
**clinical seizures**	focal seizures, onset age 7 years, frontal lobe semiology, well controlled on CBZ since age 16 years	single convulsive seizure at age 5 years; focal seizures from age 9 described as epigastric sensation, staring, oral and hand automatisms	None	collapsing episodes suspicious for seizures without clear seizure correlate on EEG	collapsing episodes of unknown etiology
**EEG**	anterior spike-wave complexes	initially normal, focal seizures from right temporal region at age 19 years	no epileptiform features	bilateral temporal sharp waves (awake), centro-temporal spikes in sleep	normal
**cognitive difficulties**	FSIQ 55	early language and reading delay but attended college (did not graduate)	borderline motor delay walked 18 months; words 3 years; later difficulty with comprehension and executive function	none reported	none reported
**dysmorphic features**	bilateral clinodactyly and pes cavus	none reported	none reported	none	none
**other clinical features**	none reported	migraine headaches	none reported	collapse episodes captured on video monitoring and determined not to be seizures; family history of psychiatric disease	collapsing episode of unknown etiology, psychiatric disease
