# Supplementary material for: De novo and inherited private variants in MAP1B in periventricular nodular heterotopia
Source: PLoS Genet. 2018 May 8;14(5):e1007281. doi: 10.1371/journal.pgen.1007281 (PMC5965900; doi:10.1371/journal.pgen.1007281)
Supplement: S5 Table — (PDF) [file pgen.1007281.s011.pdf]

S5 Table. PVNH genes prioritized in the leave one out analysis across the three datasets

Gene	Miller	Kang	Colantuoni
AKT3	1	1	0
FAT4	1	1	0
MAP1B	1	1	0
INTS8	1	0	0
DCHS1	1	0	0
NEDD4L	1	1	0
FLNA	0	1	0
ARFGEF2	0	0	0
MCPH1	0	0	0
RAPGEF2	1	1	0
MLLT4	1	0	0
EML1	1	1	0
KATNB1	0	1	0
CDH2	0	0	0
