# Supplementary material for: De novo and inherited private variants in MAP1B in periventricular nodular heterotopia
Source: PLoS Genet. 2018 May 8;14(5):e1007281. doi: 10.1371/journal.pgen.1007281 (PMC5965900; doi:10.1371/journal.pgen.1007281)
Supplement: S7 Table — (PDF) [file pgen.1007281.s013.pdf]

S7 Table. Control cohort composition

phenotype	number of individuals
amyotrophic lateral sclerosis	2631
autoimmune disease	6
bone disease	4
cardiovascular disease	68
neuropsychiatrically-normal control	3429
control with mild neuropsychiatric disease (depression, anxiety, etc.)	13
healthy family member	3523
infectious disease	377
kidney and urological disease	2541
liver disease	90
obsessive compulsive disorder	109
ophthalmic disease	11
pulmonary disease	396
Total	***13198***
