# Supplementary material for: De novo and inherited private variants in MAP1B in periventricular nodular heterotopia
Source: PLoS Genet. 2018 May 8;14(5):e1007281. doi: 10.1371/journal.pgen.1007281 (PMC5965900; doi:10.1371/journal.pgen.1007281)
Supplement: S9 Table — (PDF) [file pgen.1007281.s015.pdf]

S9 Table. Overview of transcriptomic datasets used for co-expression analyses.

Brain transcriptomic datasets	Number of transcripts evaluated in the published study	Regions evaluated	Individuals included (from 3-38 pcw)	Total number of specimens used in Brain CoX (from 3-38 pcw)	Total number of transcripts used in Brain CoX (from 3-38 pcw)
Kang et al.[3]	18,708	Frontal cortex, parietal cortex, temporal cortex, occipital cortex, hippocampus, cerebellum, thalamus	257 samples in relevant time period<br>4 female/ 6 male	1329	17,776
Miller et al.[4]	62,000	25 areas of the developing neocortex targeted	3 females/1 male	319	33,043
Colantunoi et al.[5]	49,152	dorsolateral prefrontal cortex	13 from the relevant time period (5 females and 8 males)	266	20,373
