# Supplementary material for: De novo and inherited private variants in MAP1B in periventricular nodular heterotopia
Source: PLoS Genet. 2018 May 8;14(5):e1007281. doi: 10.1371/journal.pgen.1007281 (PMC5965900; doi:10.1371/journal.pgen.1007281)
Supplement: S10 Table — (PDF) [file pgen.1007281.s016.pdf]

S10 Table. Features used in the predictive model and their relative influence in the *de novo* variant confirmation model

Feature id number	Variant level feature	Additional details	Relative influence
1	#reads_var_proband	proband: # reads supporting the variant	0.5595
2	#reads_proband	proband: total # reads at varint site	0.2736
3	#reads_var_father	father: # reads supporting the variant	0.0471
4	#reads_total_father	father: total # reads at varint site	0.0838
5	variant/total_readratio_father	father: # reads supporting the variant/total # reads at varint site	0.0984
6	#reads_var_mother	mother: # reads supporting the variant	0.0008
7	#reads_total_mother	mother: total # reads at varint site	0.2710
8	variant/total_readratio_mother	mother: # reads supporting the variant/total # reads at varint site	0.0016
9	binomial_p () - from RAW	P-value reflecting the probability of seeing the observed number of reads supporting variant in the proband out of the total number of reads assuming a heterozygous variant with an expected read ratio of 1:1 (variant:reference)	0.4015
10	FS (<=60)	de novo variants were flagged if the Fishers Strand score (GATK) was <=60	0.2566
11	HaplotypeScore (<=13)	de novo variants were flagged if the Haplotype score (GATK) was <=13	0.2256
12	MQ (>=40)	de novo variants were flagged if the Mapping Quality score (GATK) was >=40	2.9749
13	MQRankSum (>=-12.5)	de novo variants were flagged if the Mapping Quality Rank Sum (GATK) was <=-12.5	0.1195
14	QD (>=2)	de novo variants were flagged if the Quality of Depth score (GATK) was >=2	64.7010
15	ReadPosRankSum (>=-8)	de novo variants were flagged if the Read Position Rank Sum (GATK) was >=-8	0.5381
16	passQC	yes, if variant passed all feature ids 10-15; no, if variant failed any one of feature ids 10-15	0.0167
17	EXAC_maf	minor allele frequency in EVS database	1.1638
18	IGMcontrol_maf	minor allele frequency in 13,198 IGM controls	14.8001
19	in_controls (IGM or EVS/EXAC)	yes, if minor allele frequency>0 for feature ids 17 or 18; no if minor allele frequency =0 for feature ids 17 or 18	0.4296
20	Xchr	yes if the variant is located on the X chromosome; no if the variant is not located on the X chromosome	0.0092
21	Proband_PL_AA	Normalized Phred-scaled likelihood score (GATK) that the proband is not homozygous reference	7.8897
22	Father_PL_AB (lower value means more likely that hom-ref genotype wrong)	Normalized Phred-scaled likelihood score (GATK) that the proband is not heterozygous	0.0878
23	Mother_PL_AB (lower value means more likely that hom-ref genotype wrong)	Normalized Phred-scaled likelihood score (GATK) that the proband is not heterozygous	0.1599
	Individual level feature		
24	sex	sex of the proband	0.0149
25	kit	exome capture kit	0.0020
26	seqsite	site where sequencing was performed	0.0000
27	total number of de novo mutations called	total number of unfiltered de novo variants called in the proband	0.0641
28	trio level callable real estate	the percent of the CCDS regions (v14) that were sequenced at least 10-fold in mother, child and proband	0.1741
29	MeanMedianCovRatio	proband: ratio of mean to median coverage across the exome	0.1376
30	number of snvs in callable realestate	proband: number of single nucleotide variants in the callable realestate	0.0134
31	coverage	proband: average coverage across the exome	0.1065
32	pcrdup	proband: raction of reads thought to arise from PCR duplicates in the sequencing output	0.0097
33	%readsaligned	proband: percent of sequencing reads that were sucessfully aligned	0.0253
34	contamination_proband	proband: percent of contamination (VerifyBamID)	0.0299
35	titv_proband	proband: ratio of transitions to transversions among variant calls from exome sequence data	0.0154
36	dggapoverlap_proband	proband: % of overlap of variant calls with dbGAP variant calls	0.2870
37	MeanMedianCovRatio_mother	mother: ratio of mean to median coverage across the exome	0.1279
38	number of snvs in callable realestate_mother	mother: number of single nucleotide variants in the callable realestate	0.0519
39	coverage_mother	mother: average coverage across the exome	0.1459
40	pcrdup_mother	mother: raction of reads thought to arise from PCR duplicates in the sequencing output	0.0066
41	%readsaligned_mother	mother: percent of sequencing reads that were sucessfully aligned	0.0450
42	contamination_mother	mother: percent of contamination (VerifyBamID)	0.4457
43	titv_mother	mother: ratio of transitions to transversions among variant calls from exome sequence data	0.6758
44	dggapoverlap_mother	mother: % of overlap of variant calls with dbGAP variant calls	0.0314
45	MeanMedianCovRatio_father	father: ratio of mean to median coverage across the exome	0.0459
46	number of snvs in callable realestate_father	father: number of single nucleotide variants in the callable realestate	0.0165
47	coverage_father	father: average coverage across the exome	0.0709

48	pcrdup_father	father: raction of reads thought to arise from PCR duplicates in the sequencing output	0.0031
49	%readsaligned_father	father: percent of sequencing reads that were sucessfully aligned	0.0266
50	contamination_father	father: percent of contamination (VerifyBamID)	0.0374
51	titv_father	father: ratio of transitions to transversions among variant calls from exome sequence data	0.0076
52	dggapoverla_father	father: % of overlap of variant calls with dbGAP variant calls	0.0299
53	ethnicity_proband	ethinicity of proband	0.0066
54	ethnicity_mother	ethinicity of mother	0.0057
55	ethnicity_father	ethinicity of father	0.0005
	**Individual level feature**		
56	in repeat region (UCSC defined)	yes/no if variant is located in a repetative region; regions defined in: Smit AFA, Hubley R, Green P. RepeatMasker Open-3.0. http://www.repeatmasker.org. 1996-2010.	0.0017
57	segdup (UCSC)	yes/no if variant is located in a segmental duplication; regions defined in: PMID: 11381028 and PMID: 12169732	0.5279
58	simple repeat (UCSC)	yes/no if variant is located in a segmental duplication; PMID: 9862982	0.8156
59	GC percent (across 100-bp window with snv in the middle)	GC percent of 101-bp window (variant with 50-bps flanking)	0.1524
60	trinucleotide mutation rate	trinucleotide based mutation rate estimate PMC1852724, PMC4485564	0.7318
