# Supplementary material for: De novo and inherited private variants in MAP1B in periventricular nodular heterotopia
Source: PLoS Genet. 2018 May 8;14(5):e1007281. doi: 10.1371/journal.pgen.1007281 (PMC5965900; doi:10.1371/journal.pgen.1007281)

S1 Figure. Likelihood surface representing the genetic architecture of periventricular nodular heterotopia

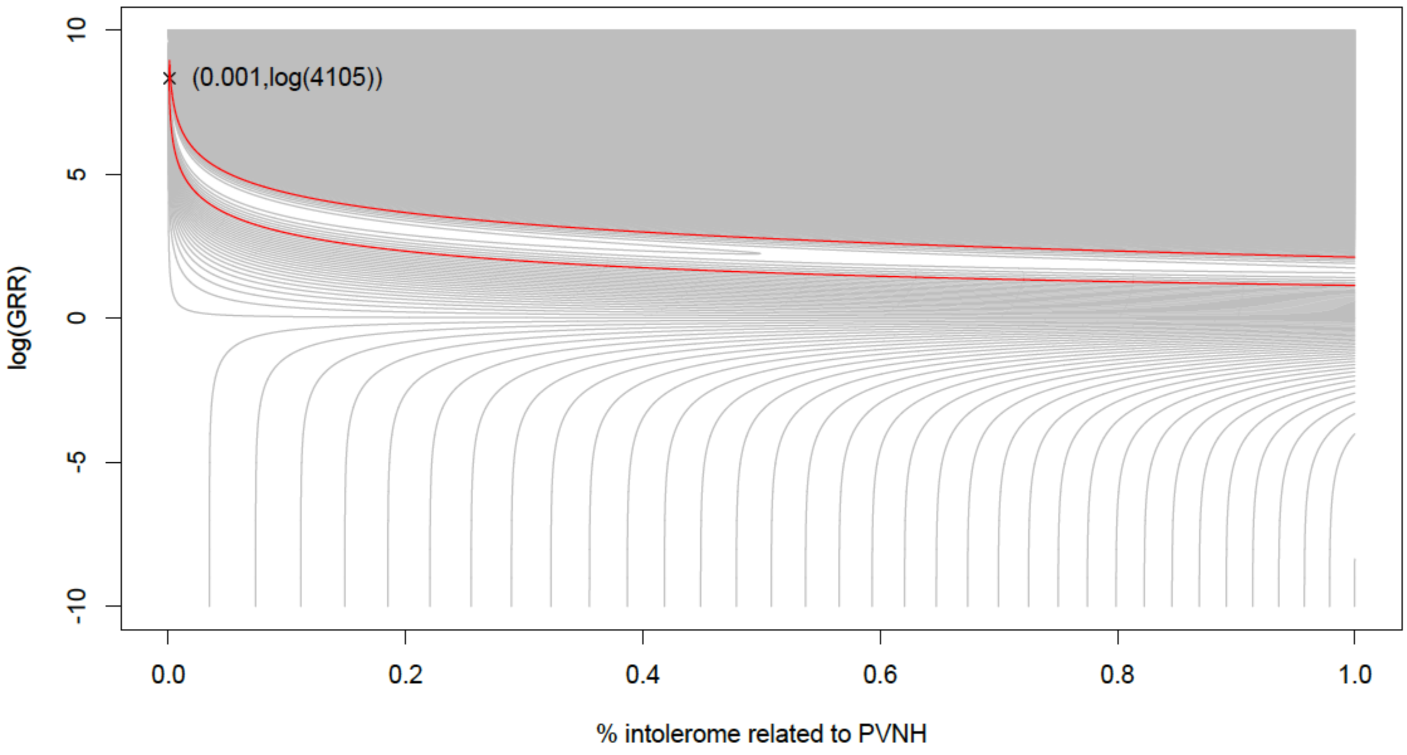

Supplement: S1 Fig — (PDF) [file pgen.1007281.s017.pdf]
