# Supplementary material for: De novo and inherited private variants in MAP1B in periventricular nodular heterotopia
Source: PLoS Genet. 2018 May 8;14(5):e1007281. doi: 10.1371/journal.pgen.1007281 (PMC5965900; doi:10.1371/journal.pgen.1007281)

S3 Figure. Quantile-quantile plot for gene-level association tests interrogating (A) LoF and “probably damaging” (Polyphen-2) missense variants, and (B) synonymous variants.

A.

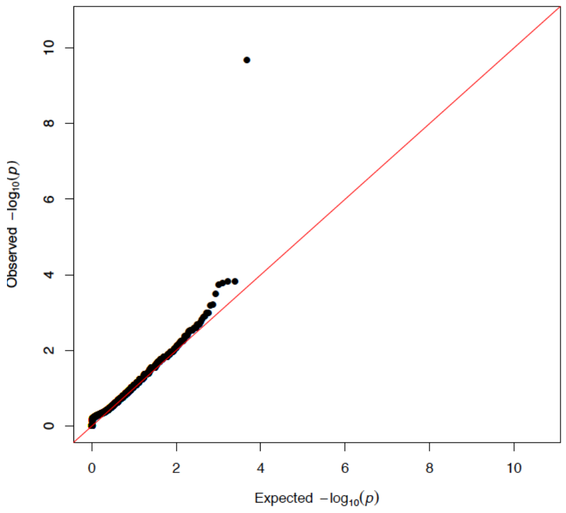

B.

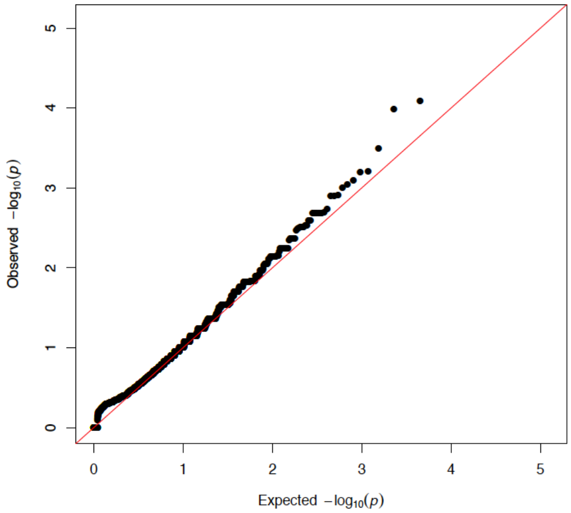

Supplement: S3 Fig — Quantile-quantile plot for gene-level association tests interrogating (A) LoF and “probably damaging” (Polyphen-2) missense variants, and (B) synonymous variants. (PDF) [file pgen.1007281.s019.pdf]
