# Supplementary material for: De novo and inherited private variants in MAP1B in periventricular nodular heterotopia
Source: PLoS Genet. 2018 May 8;14(5):e1007281. doi: 10.1371/journal.pgen.1007281 (PMC5965900; doi:10.1371/journal.pgen.1007281)

S4 Figure. Brain MRI of subjects with LoF *MAP1B* variants.

The left image is coronal T1 inversion recovery (pvhit1238Pbti1), the middle image is coronal T2-weighted (pvhnd29281lw1) and the right image is axial T2 weighted (pvhcw12701bvi1). All images show periventricular nodular grey matter heterotopia maximal in the frontal regions (arrows). No polymicrogyria was seen in subjects pvhit1238Pbti1 or pvhnd29281lw1. There was possible right insular polymicrogyria in pvhcw12701bvi1, but the available images were not of sufficient quality to be conclusive. MRI image for fourth patient is provided in Figure 3.

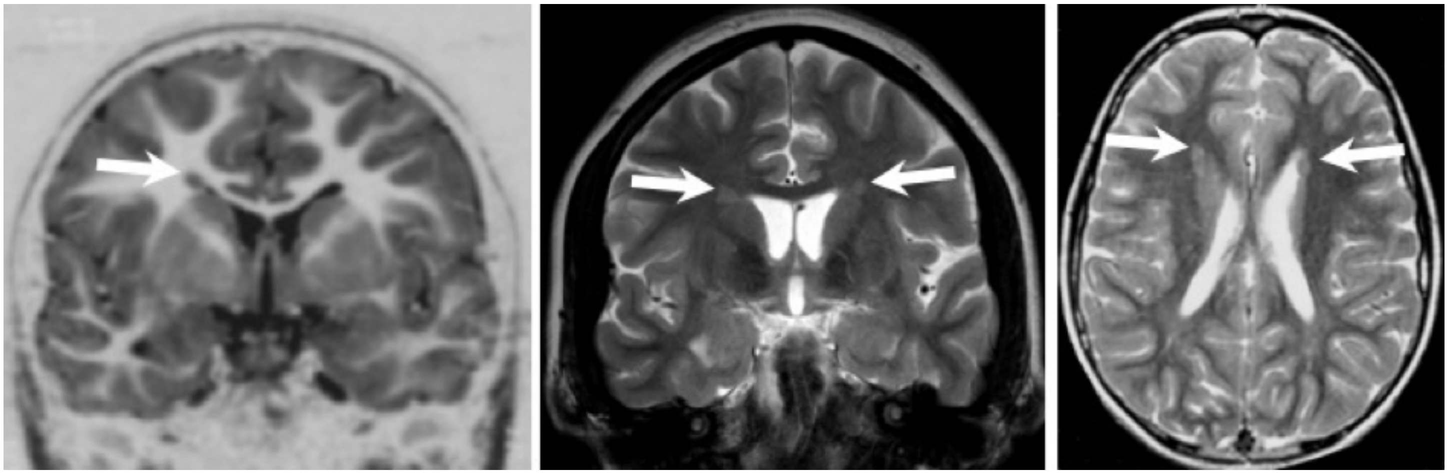

Supplement: S4 Fig — The left image is coronal T1 inversion recovery (pvhit1238Pbti1), the middle image is coronal T2-weighted (pvhnd29281lw1) and the right image is axial T2 weighted (pvhcw12701bvi1). All images show periventricular nodular grey matter heterotopia maximal in the frontal regions (arrows). No polymicrogyria was seen in subjects pvhit1238Pbti1 or pvhnd29281lw1. There was possible right insular polymicrogyria in pvhcw12701bvi1, but the available images were not of sufficient quality to be conclusive. MRI image for fourth patient is provided in Fig 3. (PDF) [file pgen.1007281.s020.pdf]
