# Supplementary material for: De novo and inherited private variants in MAP1B in periventricular nodular heterotopia
Source: PLoS Genet. 2018 May 8;14(5):e1007281. doi: 10.1371/journal.pgen.1007281 (PMC5965900; doi:10.1371/journal.pgen.1007281)

S5 Figure. Cumulative proportion of correlation coefficients for 14 genes implicated in PVNH compared to 1000 randomly selected sets of 14 genes.

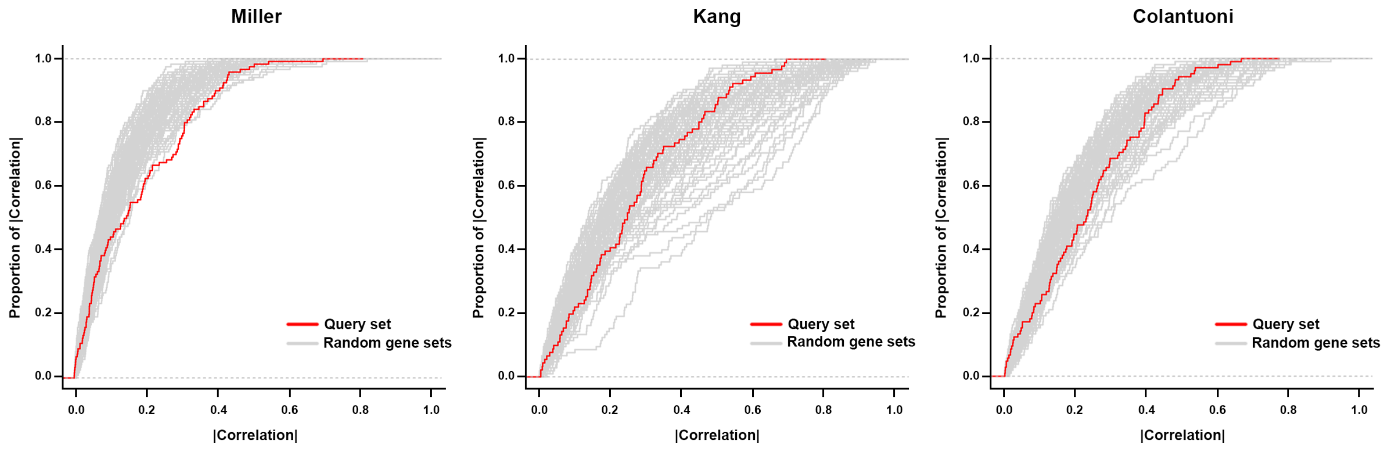

Supplement: S5 Fig — (PDF) [file pgen.1007281.s021.pdf]
