# Supplementary material for: De novo and inherited private variants in MAP1B in periventricular nodular heterotopia
Source: PLoS Genet. 2018 May 8;14(5):e1007281. doi: 10.1371/journal.pgen.1007281 (PMC5965900; doi:10.1371/journal.pgen.1007281)
Supplement: S6 Fig — Pairwise Pearson’s correlations between prioritized genes harboring de novo mutations based on co-expression profiles and the 8 human query genes through each time period analyzed and presented across each row. Patterns of positive (+1) and reciprocal (-1) co-regulatory interactions are represented as blue and red squares, respectively. Data was derived from the Miller and Kang transcriptomic datasets with the time points in which the data was derived being separated into specific periods as indicated in by the value below each point; 1, 4-8pcw; 2, 8-10pcw; 3, 10-13pcw; 4, 13-16pcw; 5, 16-19pcw; 6, 19-24pcw; 7, 24-38pcw; 8, birth-6M; 9, 6M-1Y; 10, 1-6Y; 11, 6-12Y; 12, 12-20Y; 13, 20-40Y; 14, 40-60Y; 15, 60Y+. pcw, weeks post conception; M, months after birth; Y, years after birth. (PDF) [file pgen.1007281.s022.pdf]

S6 Figure. Correlation matrices for prioritized genes compared to each known human PVNH query gene individually and across different periods of brain development.

Pairwise Pearson's correlations between prioritized genes harboring de novo mutations based on co-expression profiles and the 8 human query genes through each time period analyzed and presented across each row. Patterns of positive (+1) and reciprocal (-1) co-regulatory interactions are represented as blue and red squares, respectively. Data was derived from the Miller and Kang transcriptomic datasets with the time points in which the data was derived being separated into specific periods as indicated in by the value below each point; 1, 4-8pcw; 2, 8-10pcw; 3, 10-13pcw; 4, 13-16pcw; 5, 16-19pcw; 6, 19-24pcw; 7, 24-38pcw; 8, birth-6M; 9, 6M-1Y; 10, 1-6Y; 11, 6-12Y; 12, 12-20Y; 13, 20-40Y; 14, 40-60Y; 15, 60Y+. pcw, weeks post conception; M, months after birth; Y, years after birth.

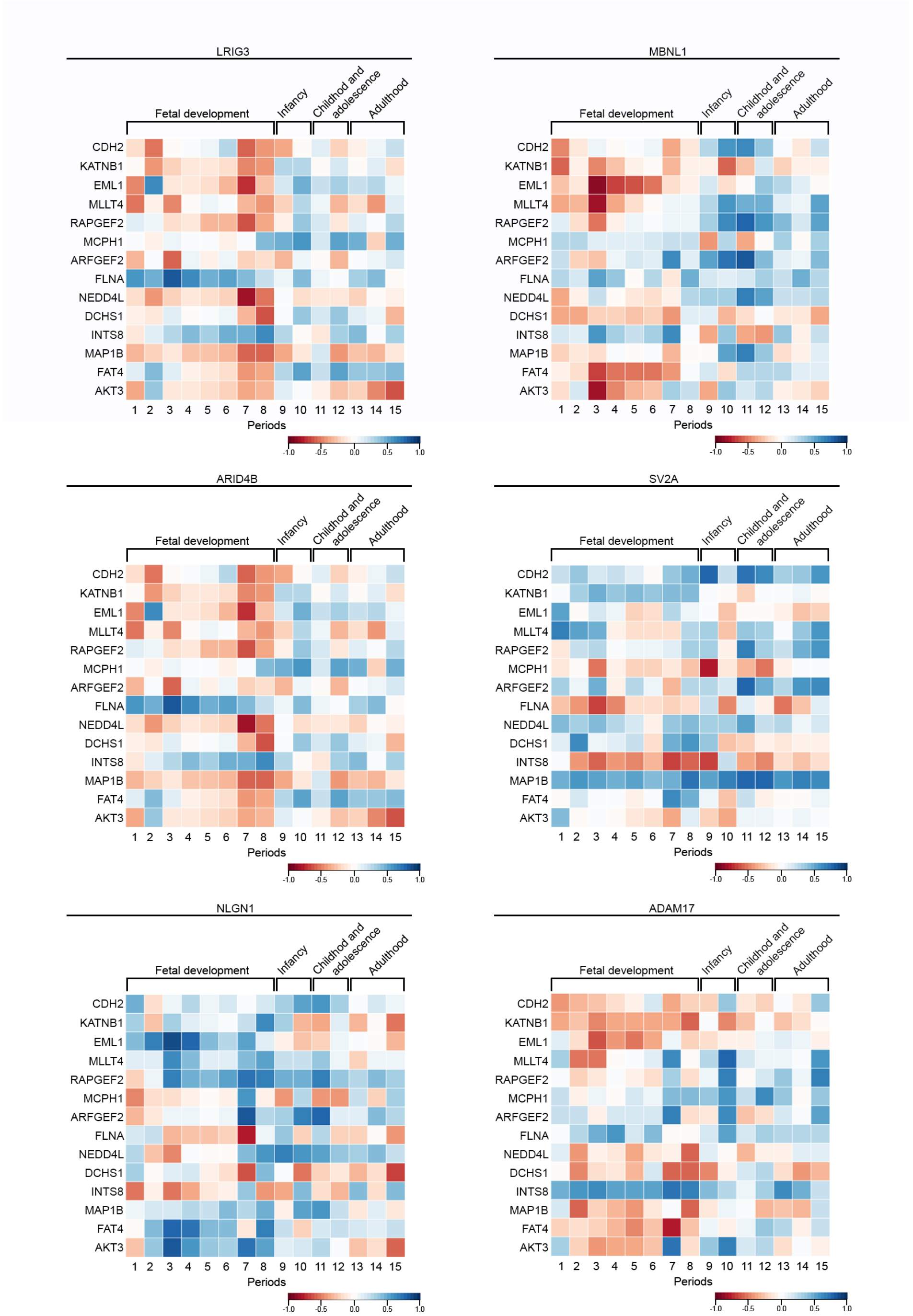

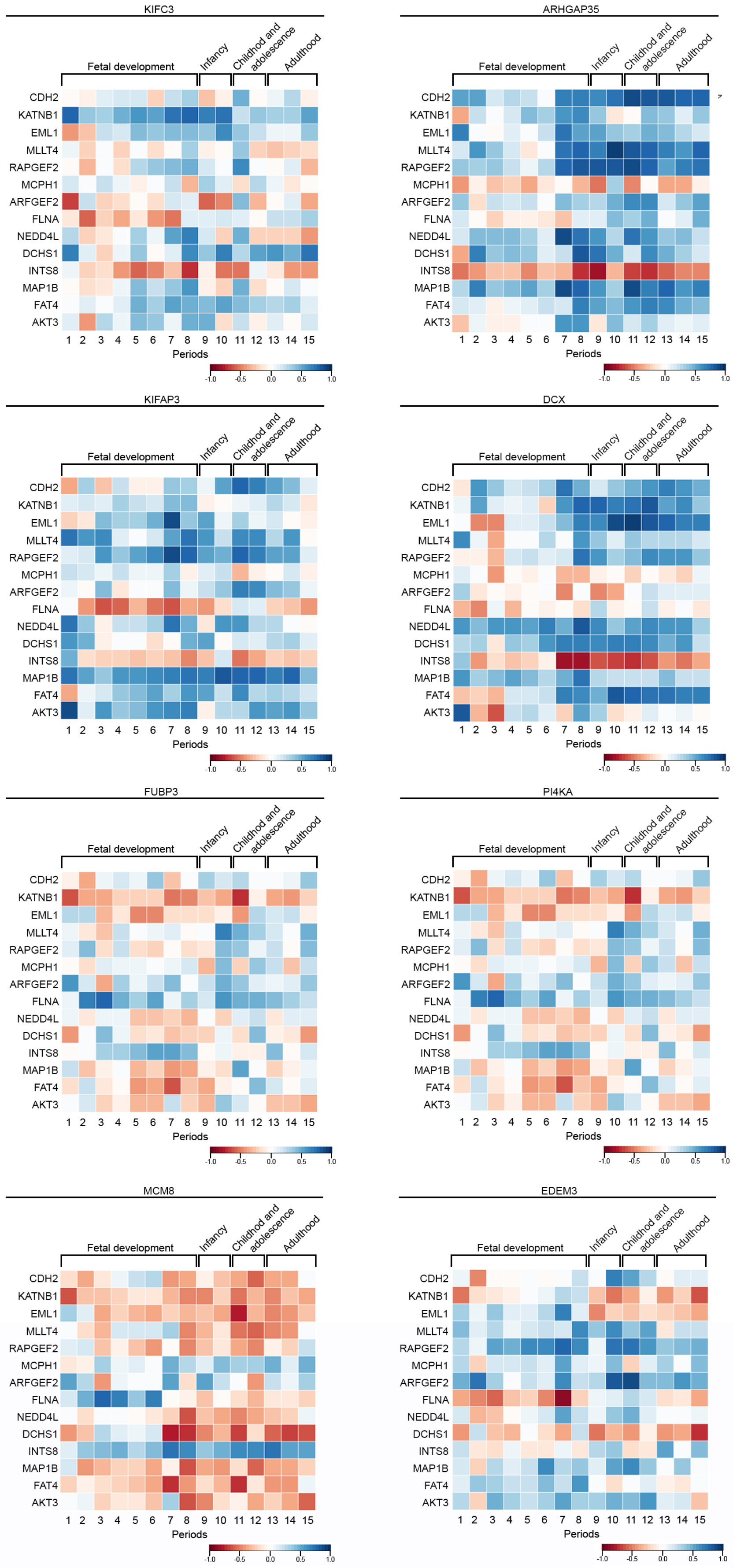
KIFC3
ARHGAP35
KIFAP3
DCX
FUBP3
PI4KA
MCM8
EDEM3
Fetal development
Infancy
Childhod and adolescence
Adulthood
CDH2
KATNB1
EML1
MLLT4
RAPGEF2
MCPH1
ARFGEF2
FLNA
NEDD4L
DCHS1
INTS8
MAP1B
FAT4
AKT3
1 2 3 4 5 6 7 8 9 10 11 12 13 14 15
Periods
-1.0 -0.5 0.0 0.5 1.0
